# *ACT-Discover*: identifying karyotype heterogeneity in pancreatic cancer evolution using ctDNA

**DOI:** 10.1186/s13073-023-01171-w

**Published:** 2023-04-20

**Authors:** Ariana Huebner, James R. M. Black, Francesca Sarno, Roberto Pazo, Ignacio Juez, Laura Medina, Rocio Garcia-Carbonero, Carmen Guillén, Jaime Feliú, Carolina Alonso, Carlota Arenillas, Ana Belén Moreno-Cárdenas, Helena Verdaguer, Teresa Macarulla, Manuel Hidalgo, Nicholas McGranahan, Rodrigo A. Toledo

**Affiliations:** 1 Cancer Research UK Lung Cancer Centre of Excellence, University College London Cancer Institute, London, UK; 2 Cancer Genome Evolution Research Group, Cancer Research UK Lung Cancer Centre of Excellence, University College London Cancer Institute, London, UK; 3 Cancer Evolution and Genome Instability Laboratory, The Francis Crick Institute, London, UK; 4 Peaches Biotech, Madrid, Spain; 5 Hospital Universitario Miguel Servet, Zaragoza, Spain; 6 Hospital Universitario de Fuenlabrada, Madrid, Spain; 7 IBIMA, Virgen de La Victoria, Malaga, Spain; 8 Hospital Universitario 12 de Octubre, Madrid, Spain; 9 Hospital Universitario Ramón Y Cajal, Madrid, Spain; 10 Hospital Universitario La Paz, Madrid, Spain; 11 Vall d’Hebron Institute of Oncology (VHIO), Barcelona, Spain; 12 Centro de Investigación Biomédica en Red de Cáncer (CIBERONC), Madrid, Spain; 13 Vall d’Hebron University Hospital, Barcelona, Spain; 14 Weill Cornell Medicine, New York, USA

**Keywords:** Pancreatic cancer, Tumour evolution, Intra-tumour heterogeneity, ctDNA, Copy number, cfDNA, Circulating tumour DNA, Cell-free DNA

## Abstract

**Background:**

Liquid biopsies and the dynamic tracking of somatic mutations within circulating tumour DNA (ctDNA) can provide insight into the dynamics of cancer evolution and the intra-tumour heterogeneity that fuels treatment resistance. However, identifying and tracking dynamic changes in somatic copy number alterations (SCNAs), which have been associated with poor outcome and metastasis, using ctDNA is challenging. Pancreatic adenocarcinoma is a disease which has been considered to harbour early punctuated events in its evolution, leading to an early fitness peak, with minimal further subclonal evolution.

**Methods:**

To interrogate the role of SCNAs in pancreatic adenocarcinoma cancer evolution, we applied whole-exome sequencing of 55 longitudinal cell-free DNA (cfDNA) samples taken from 24 patients (including 8 from whom a patient-derived xenograft (PDX) was derived) with metastatic disease prospectively recruited into a clinical trial. We developed a method, Aneuploidy in Circulating Tumour DNA (ACT-Discover), that leverages haplotype phasing of paired tumour biopsies or PDXs to identify SCNAs in cfDNA with greater sensitivity.

**Results:**

SCNAs were observed within 28 of 47 evaluable cfDNA samples. Of these events, 30% could only be identified by harnessing the haplotype-aware approach leveraged in ACT-Discover. The exceptional purity of PDX tumours enabled near-complete phasing of genomic regions in allelic imbalance, highlighting an important auxiliary function of PDXs. Finally, although the classical model of pancreatic cancer evolution emphasises the importance of early, homogenous somatic events as a key requirement for cancer development, ACT-Discover identified substantial heterogeneity of SCNAs, including parallel focal and arm-level events, affecting different parental alleles within individual tumours. Indeed, ongoing acquisition of SCNAs was identified within tumours throughout the disease course, including within an untreated metastatic tumour.

**Conclusions:**

This work demonstrates the power of haplotype phasing to study genomic variation in cfDNA samples and reveals undiscovered intra-tumour heterogeneity with important scientific and clinical implications. Implementation of ACT-Discover could lead to important insights from existing cohorts or underpin future prospective studies seeking to characterise the landscape of tumour evolution through liquid biopsy.

**Supplementary Information:**

The online version contains supplementary material available at 10.1186/s13073-023-01171-w.

## Background

Pancreatic cancer is the seventh most common cause of cancer-related death worldwide [1]. Despite the increasing incidence of this disease, effective treatments are lacking, and the prognosis for patients with pancreatic cancer remains exceptionally poor, with approximately 70% of patients dying within 1 year of diagnosis [2].

There is therefore an urgent need to characterise vulnerabilities and evolutionary dependencies that might represent therapeutic targets in this disease. There is an ongoing debate about the contribution of subclonal alterations to the pathogenesis and progression of this disease. Genetic point mutations in this tumour type, particularly within the genes *KRAS*, *TP53*, *SMAD4* and *CDKN2A*, are predominantly, though not exclusively, clonal [3]. It has therefore been suggested that when considering point mutations alone, subclonal selection might be limited in pancreatic adenocarcinoma [4]. However, such studies have typically not focused on somatic copy number alterations (SCNAs), that might act as a substrate for subclonal selection in tumours [5, 6].

To perform related analyses and resolve the evolutionary status and importance of SCNAs, it is for the most part necessary to have multiple tissue samples over space or time [7, 8]. However, it can be difficult to obtain these samples from patients with pancreatic cancer. This is due to the typically low tumour content of biopsy specimens in this disease as well as the difficulty of biopsy in patients with recurrent disease who are often profoundly unwell.

Non-invasive liquid biopsies, from which circulating tumour DNA (ctDNA) can be isolated, represent an important alternative strategy for gaining insight into tumour evolutionary dynamics over time, although existing approaches typically study point mutations, rather than SCNAs [9–11]. Similarly, obtaining patient-derived xenografts (PDXs) of tissue biopsies, which provide very pure near-replicas of patient tumours, can provide a useful model to further understand cancer genomics while simultaneously overcoming the challenge of low tumour content in this disease and thereby enabling analysis of SCNAs [12, 13].

Here, we characterise the evolution of late-stage pancreatic cancer in a prospectively recruited, longitudinal patient cohort. Using *ACT-Discover*, which leverages matched 'germline' blood DNA, biopsy specimens, PDXs and/or ctDNA, we reveal the ongoing acquisition of subclonal alterations that would not have been identified within a single snapshot in space and time. Furthermore, we utilise *ACT-Discover* to highlight instances of mirrored subclonal allelic imbalance in evolving cancer, suggestive of ongoing chromosomal instability and karyotype remodelling in advanced tumours. This work highlights important additional uses of liquid biopsy to understand tumour evolution.

## Methods

### Ethics committee approval

The study was approved by the Comité Ético de Investigación Clínica Regional de la Comunidad de Madrid (CEIC-R) and the Vall d’Hebron Institute of Research (VHIR) Ethics Committees. The research was conducted in accordance with the Declaration of Helsinki and local data protection laws. All patients were provided with written informed consent before enrolment. All data provided were anonymised in line with applicable laws and regulations.

### Patient enrolment

Twenty of these patients were enrolled on an investigator-initiating multicenter, open-label, randomised phase II clinical trial led by Dr. Hidalgo and sponsored by the Hospital Universitario de Fuenlabrada, Madrid, Spain (NCT02795650) that enrolled a total of 129 patients. Preliminary data of this trial was recently reported as an abstract [14]. This work does not report any data or result of the referred clinical trial. Additionally, 4 patients were enrolled from the Vall d’Hebron Institute of Oncology (VHIO, Barcelona, Spain). Plasma samples were collected from the patients as they enrolled to limit selection bias. Although 129 patients were enrolled, it was only possible to analyse DNA from a subset due to financial limitations (Additional file 1: Fig. S1). Overall, 87 tumour samples (including 2 primary tumour, 22 metastasis, 8 PDX and 55 cell-free DNA (cfDNA) samples) and 24 germline samples from 24 patients were whole-exome sequenced. Of these, a total of 75 tumour samples (including 2 primary tumour, 18 metastasis, 8 PDX and 47 cfDNA samples) from 19 patients were evaluable and used for subsequent analyses.

### Sample extraction and whole-exome sequencing

DNA was extracted from tumour tissue using the QIAGEN DNeasy Blood and Tissue kit (Qiagen, Germany); 100 µl of peripheral blood was used for extraction of germline DNA using the QIAGEN DNeasy Blood and Tissue kit (Qiagen, Germany) according to the manufacturer’s recommendation and quantified using the Qubit kit dsDNA HS (high sensitivity) Assay Kit (Thermo Fisher Scientific, USA). The Illumina HiSeq platform was used to perform whole-exome sequencing on metastatic biopsies and PDX samples. Metastatic samples were sequenced to a median depth of 103 × . Blood samples were collected in Streck BCT tubes, and cfDNA from 1 mL of plasma was extracted using the QIAamp Circulating Nucleic Acid Kit (Qiagen, Germany). Genomic libraries were generated using 10 to 15 ng of cfDNA and the ThruPLEX DNA or Plasma-seq Kit (Rubicon Genomics Inc., USA; now commercialised as SMARTer Thru-PLEX plasma-seq kit by Takara Bio, Otsu, Japan). Quality control of libraries was carried out in Tapestation (Agilent, USA), and amplified profiles of ~ 300 bp were considered for downstream analysis. The capture of the genomic coding regions for whole-exome sequencing (WES) was performed using the SureSelect Human All Exon V5 or V6 kits (Agilent, USA) and the aimed sequencing output was 12 Gb (100 ×) for germline DNA and 36 Gb (300 ×) for tumour and cfDNA. Sequencing was carried out in HiSeq or Novaseq Illumina sequencers platforms.

All sequencing data have been deposited in the European Genome–Phenome Archive under accession number EGAS00001007077.

### Patient-derived xenografts

Animal work was performed according to the protocols approved by the Ethical Committees for the Use of Experimental Animals at Hospital Universivario de Fuenlabrada, animal facility centro apoyo tecnológico (CAT) of the Universidad Rey Juan Carlos (URJC) and the Vall d’Hebron Institute of Oncology (VHIO), Spain.

#### Tumour samples

Hepatic metastatic samples from core needle biopsies were freshly collected into RPMI medium and added with pen/strep 1:100 v/v. Tumour samples, free from fat and necrotic tissue, were cut into small pieces of 3 x 3 x 3 mm and embedded in Matrigel (Corning Matrigel Basement Membrane Matrix, #354234). Five- to 6-week-old female Nod Scid Gamma mice (NSG) [strain NOD.Cg-*Prkdc*^*scid*^* Il2rg*^*tm1Wji*^*/*SzJ] provided by Charles River were anaesthetised using isoflurane gas anaesthesia and administered with buprenorphine dosed at 0.2 mg/kg. Subsequently, each piece of the tumour sample was implanted subcutaneously, by using an 18-gauge trocar, in one flank of the lower back of the mice. Due to the scarcity of the tumour samples proceeding from liver biopsies, in this first phase (F1, engraftment phase), only 2 or 3 NSG were used to be implanted.

#### Establishment of xenograft

Five- to 6-week-old female athymic nude-Foxn1 (nude/nude), mice [strain Crl:UN(NCr)-Foxn1 < nu >], provided by Charles River, were anaesthetised as above-mentioned. Xenografts obtained from F1 were excised, and a part was cut into small 3 × 3 × 3 mm fragments and then implanted subcutaneously in both mice flanks, in a group of 5–8 mice (F2, expansion phase). The remaining part of the xenograft was cryopreserved and/or processed for future biological studies. Before the tumours from F2 reached a size of 1500 mm^3^, they were excised, cut into 3 × 3 × 3 mm fragments and finally implanted into the experimental cohorts of mice that were treated with the drugs (F3 and successive).

Mice from all phases were monitored during the study and were humanely sacrificed after the appearance of any of the following: tumour size approaching 1500 mm^3^, loss of body weight, lethargy, dyspnea and/or pain.

### Variant calling (and pipeline overview)

The samples were aligned to the reference human genome (hg19) using Burrows-Wheeler algorithm (BWA) [15]. The median coverage of the samples (excluding the PDX samples) was 103 (range 38–201) and the median coverage of the PDX samples was 210 (range 49-424). The median coverage of the germline samples was slightly lower than the median coverage of the tumour samples (median of 94 and 107, respectively).

The PDX samples were aligned to the reference human genome (hg19) as well as to the reference mouse genome (mm10) using BWA. Subsequently, bamcmp [16] was used to categorise reads into human only, human better, mouse only and mouse better. All reads mapping better to the human reference (human only and human better) were combined and used in downstream processing and analysis.

The samples were processed using a modified version of the pipeline described in Jamal-Hanjani et al. [17]. In brief, VarScan2 [18] and MuTect [19] were used to identify somatic variants and subsequently additional filtering was performed. A mutation was passed if the variant allele frequency (VAF) was greater than 2% and was called by both MuTect and VarScan2. If the mutation was only called by VarScan2, a VAF of 5% was required. In both cases, a somatic *p*-value of ≤ 0.01 was required for the mutation to be called by VarScan2. Furthermore, a sequencing depth ≥ 10 at the mutated position was required in every sample. As a rule, > 5 reads were needed to support the variant call; however, if there were ≥ 3 reads supporting the variant call and the mutation was called by both VarScan2 and MuTect, the mutation was passed. Finally, the number of reads supporting the variant in the germline needed to be < 3 with a VAF ≤ 1%. Somatic variants were annotated as driver mutations as described in Jamal-Hanjani et al. [17] using a list of pan-cancer and pancreatic cancer genes curated by Bailey et al. [20]. 

Initial purity and ploidy solutions were obtained from the implementation of ASCAT [21] with manual review. Of the cfDNA samples, 6 samples had sufficient tumour purity to estimate copy number aberrations while the remaining 49 had very low tumour purity (estimated as < 5%). Six metastasis biopsies (from PAN103, PAN110, PAN114, PAN119, PAN121, PAN122) as well as one sample of the primary tumour obtained from PAN103 had < 5% tumour content. The remaining 16 metastasis biopsies and the remaining sample of the primary tumour from PAN103, as well as the 8 PDX samples, had sufficiently high tumour content (> 15%). The mean purity of these 31 samples was 54% (range 15–100%).

For two patients (PAN114, PAN122), no mutations from any of the samples passed filtering, therefore all samples (2 samples for PAN114, 4 samples for PAN122) from these patients were excluded from subsequent analyses. Additionally, three patients (PAN106, PAN119, PAN121) had no samples with sufficient tumour purity to determine copy number status; however, mutation data was still used for these cases. Thus, in total, 47 cfDNA, 18 metastasis and 8 PDX, as well as 2 primary tumour samples, from 19 patients were deemed evaluable and used in subsequent analyses. Notably, for eight patients (PAN103, PAN104, PAN105, PAN108, PAN109, PAN113, PAN125, PANVH1), multi-sample copy number data was available.

### Threshold analysis for lower SNV filters

For a subset of cfDNA samples (6 of 47), high tumour purity (≥ 15%) was observed. However, in the majority of cfDNA samples (41 of 47), tumour purity was below 5%. Therefore, tumour content and copy number aberrations could not be reliably estimated using ASCAT. Due to low tumour content, in most cases, de novo mutation calling only yielded a low number of mutations (median number of mutations: 3, range 0–100). In several cfDNA samples, no mutations were identified using de novo calling, while in the majority of samples (41 of 47), less than 10 mutations were called.

In order to increase the power to detect mutations in the cfDNA samples, mutations confidently called in other samples of the same patient were leveraged. However, to reduce the rate of false-positive mutations, multiple thresholds were explored. We reasoned that *bona fide* mutations in cfDNA samples should tend to be patient-specific, unless they resided in driver mutations. Conversely, artefactual mutations or sequencing noise, are more likely to be recurrent across samples from different patients. Thus, we considered which filters can be used to optimise true positive mutations at the expense of false-positive mutations.

Initially, all mutations that were confidently called in at least one sample of the patient were collated. Mutations in cancer genes that were classified as driver mutations were removed from this analysis as some overlap of such mutations can be expected. Subsequently, for each patient, read information for these high confidence mutations was extracted from the alignment files of the samples of all patients using bam-readcount (https://github.com/genome/bam-readcount). For each patient, mutations in each cfDNA sample were called from the initially passed mutations using a variant read count filter of 1, 2, 3, 5, 10 or more reads and a VAF filter of greater than 0%, 1% and 1.5%. Mutations positively identified in the cfDNA sample of interest based on these filters were subsequently explored in cfDNA samples from all patients. The proportion of mutations positively identified based on the same filters in at least one other cfDNA sample was calculated and weighed against the total number of mutations identified in the cfDNA sample of interest. The results can be seen in Additional file 1: Fig. S2.

To determine a reasonable threshold with which to positively identify high-confidence mutations in the cfDNA, the tradeoff between the total number of mutations identified and the proportion of mutations also found in at least one other cfDNA sample was considered. When using lenient filters of one or more variant read and a VAF greater than 0%, at least one mutation was identified in all cfDNA samples (median 24; range 1–124). However, in this case, more than 25% of mutations for all cfDNA samples were found in samples of other patients (median 73%; range 33–100). Conversely, when using very stringent filters whereby 10 or more variant reads and a VAF greater than 1.5% were required to call a mutation, 42 cfDNA samples had less than 5 mutations (median 2; range 0–107), but in this case, only a single mutation was found in another cfDNA sample from another patient while the remaining samples did not share mutations. Thus, while stringent filters reduce the number of shared cfDNA mutations (which we can assume predominantly reflect false-positive mutations), these also considerably reduce the number of mutations called (and thereby the number of true positives).

We observed that adopting filters such that a variant read count of 3 or more was required as well as a VAF of greater than 1% or 1.5%, provided a reasonable balance between a high number of likely true positives and a very low estimated number of false positives (Additional file 1: Fig. S3).

### SCNA rescuing

In the majority of cfDNA samples (41/47), the tumour purity was estimated as lower than 5%, meaning analysis was not possible using ASCAT [21]. However, for 19 patients, at least one tumour sample (metastasis biopsy or PDX) with purity greater than 5% was sequenced and could be leveraged for the estimation of copy number aberrations.

For each case, the segmentation as provided by ASCAT for each of the samples with tumour content > 5% was overlapped to determine the minimum consistent genomic regions across all samples. Each of these genomic regions had a number of SNPs associated with the segment, with individual B-allele frequency (BAF) estimates for each SNP as well as a segmentBAF (i.e. the median BAF across the segment) calculated by ASCAT.

For each of these minimum consistent genomic regions, the sample with the largest segmentBAF was selected, and all SNPs associated with the segment were classified as belonging to allele A or B based on the individual BAF estimates within that sample being greater or less than 0.5, respectively. The classification of SNPs was then applied to all other samples, including those with no discernible tumour content, in a given patient.

If the segmentBAF of all samples was equal to 0.5, meaning no copy number aberration was detected at that locus, the SNPs were not phased. 

To determine whether a copy number aberration was present in another sample from the same patient, comparisons were performed to test whether the distribution of SNPs from alleles A and B were significantly different in the sample of interest. For this comparison, only segments were considered where a copy number aberration was detected by ASCAT in at least one sample. A Wilcoxon test was then used to compare the BAF distributions of the SNPs belonging to alleles A and B (*p* < 0.005).

Finally, for segments with copy number aberrations, mirrored subclonal allelic imbalance (MSAI) [17] was explored. If for a given segment, the BAFs of SNPs classified as belonging to allele A, i.e. the major allele, were significantly lower than the BAFs of SNPs belonging to allele B in a given sample (one-sided Wilcoxon test, *p* < 0.01), the segment was defined as exhibiting mirrored subclonal allelic imbalance.

### Estimating tumour content in cfDNA samples

For a subset of samples in the cohort (31 samples) including 17 tissue samples as well as 6 cfDNA samples, tumour purity could be estimated using ASCAT. Additionally, for each sample, the mean VAF of ubiquitous mutations (mean clonal VAF) could be calculated. The mean clonal VAF reflects the purity of the tumour sample. Indeed, consistent with this, the two measures (mean clonal VAF and ASCAT estimated purity) were highly correlated, and using a linear model, the slope and intercept could be calculated (Additional file 1: Fig. S4). Using these values, a purity estimate for low-purity cfDNA samples could be calculated using the mean clonal VAF calculated for each sample.

### Simulating BAF profiles

For a given purity $p$ and copy number estimates of allele A $C{N}_{a}$ and B $C{N}_{b}$, the expected BAF $BA{F}_{e}$ can be calculated as [21]:

$${\mathrm{BAF}}_{e}=\frac{1-p+(p*C{N}_{b})}{2-2*p+p*(C{N}_{a}+C{N}_{b})}$$

To simulate a segment with a total of $N$ SNPs, first the number of SNPs allocated to allele B $\left(N_B\right)$ was randomly sampled from a binomial distribution $\left(n=N,\;p=0.5\right)$ and the number of SNPs allocated to allele A was set to $N-N_B$. Then, for each SNP the BAF was calculated as follows: given a purity $p$, copy number estimates of allele A $C{N}_{a}$ and B $C{N}_{b}$ the expected BAF was calculated using the equation above. The number of variant reads containing the SNP was sampled from a binomial distribution $\left(n=D,\;p=BAF_e\right)$. Finally the BAF of the SNP was calculated as the number of variant reads divided by the depth $D$. This was repeated for all SNPs of allele B and a similar approach was applied to simulate SNPs for allele A.

### Downsampling of PAN104 PDX

To simulate different effective purity samples from PAN104 PDX, the MixBAMs function from MASCoTE [22] was used with the PAN104 PDX and PAN104 germline samples as input. The BAMs created by the tool were then run through Platypus [23] in order to calculate the read counts and BAFs of all SNPs that were then used as input for *ACT-Discover.* The original PAN104 PDX sample was used as the high-purity reference to perform the SNP phasing.

### Calculating the overall classification of SCNA heterogeneity

Copy number segments that could not be phased in any sample were defined as “no SCNA detected” overall. If mirrored subclonal allelic imbalance was detected in at least one sample, the segment was classified as “MSAI” overall. If the major allele was consistent across all samples, this was defined as a “homogeneous SCNA”. If no allelic imbalance could be detected in a sample, 100 simulations of SNPs at a depth of 50, given the same copy number state, purity of the sample, and number of SNPs on the segment were performed and the outputted BAFs tested. If in at least one of the 100 simulations allelic imbalance could not be detected, the segment in the sample was defined as not confidently absent and therefore excluded from the overall classification. Only samples with confidently detected or absent events were used.

## Results

### Patient cohort

**Fig. 1:**
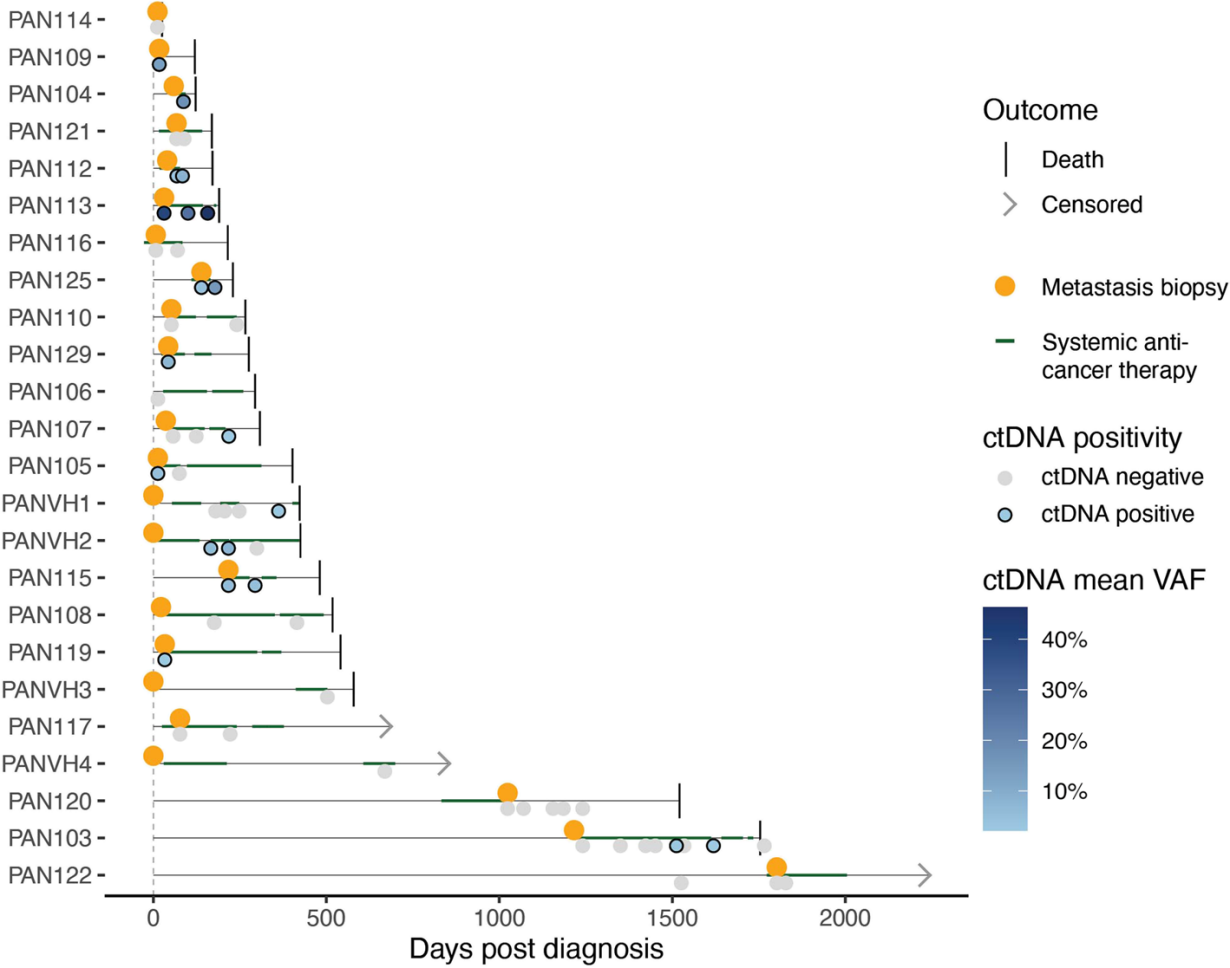
Cohort overview. Cohort of patients with metastatic pancreatic cancer. The date of metastatic biopsy relative to the time of diagnosis, systemic anti-cancer therapy, liquid biopsies and death are annotated. ctDNA - circulating tumour DNA; VAF - variant allele frequency

We assembled a prospectively recruited cohort of 24 patients with advanced pancreatic ductal adenocarcinoma who had undergone sampling of their metastatic lesion (Fig. 1, Additional file 1: Fig. S1). Three of these patients were recruited with M0 disease and subsequently relapsed, while twenty-one had M1 disease upon recruitment into the study. Twenty-one patients were followed up until death, with three patients alive at the end of the study period. One patient was lost to follow-up. The median survival from the date of biopsy of the metastatic lesion was 264 days (range 13–730 days).

Whole-exome sequencing (WES) of a metastatic biopsy specimen was performed on 22/24 patients, including one patient where, in addition to the metastasis, two regions of the primary tumour were sequenced. For eight patients, patient-derived xenograft (PDX) models were derived from the metastasis biopsy and subjected to WES, including one patient in which it was not possible to perform WES on the metastatic biopsy. Furthermore, a total of 55 cell-free DNA (cfDNA) samples were extracted, ranging from 1 to 8 (median 2) per patient.

### Genomic characteristics of pancreatic metastasis

**Fig. 2:**
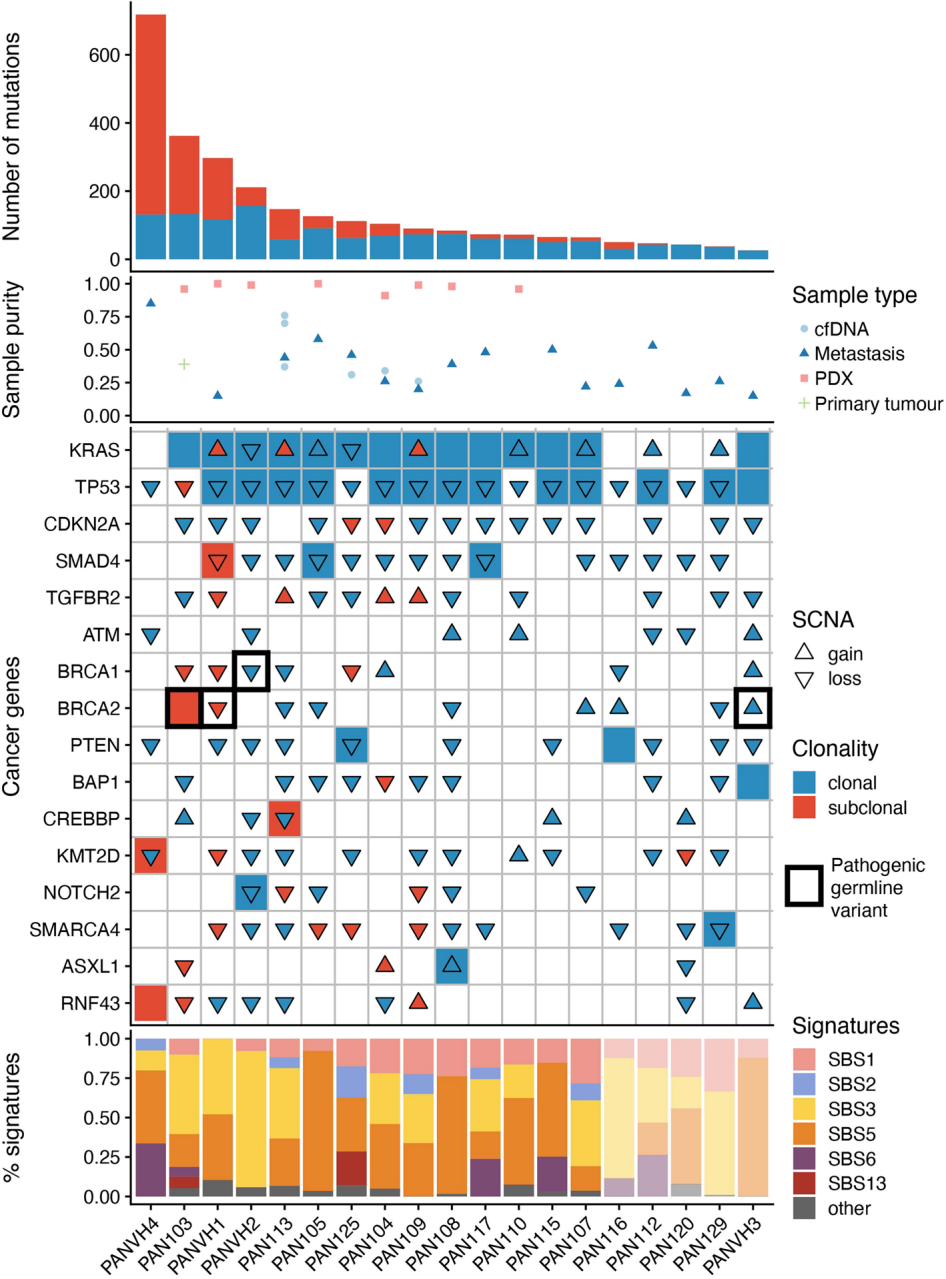
Genomic characteristics. The top panel shows the mutation load for each tumour, split into clonal mutations that are found in all samples, and subclonal mutations found only in a subset. The second panel shows the fraction of each admixed sample estimated to be derived from tumour (purity), split by whether the sample was derived from a tissue sample of primary tumour (*n* = 1), metastasis (*n* = 16), patient-derived xenograft (*n* = 8), or cfDNA (*n* = 6). Only samples with an estimated tumour purity > 5% are shown. The third panel shows the different genomic events for a set of cancer genes of interest. Mutations are represented as tiles, and somatic copy number aberrations (SCNAs) are overlaid as triangles. The colour of the tiles and triangles represents the timing of the mutations and copy number events, respectively. The final panel shows the proportion of mutations attributed to different mutational signatures for each patient. The five patients on the right of the figure, which are greyed out, do not have sufficient mutations (less than 50) to perform reliable signature deconvolution

The genomic characteristics of the evaluable patient cohort are displayed in Fig. 2. This information was gathered from the analysis of WES of metastatic biopsy samples (total *n* = 18; of which 16 had sufficient tumour content > 5%), primary tumour samples (total *n* = 2, of which 1 had sufficient tumour content > 5%), any PDX samples (*n* = 8), and cfDNA samples with sufficient tumour content (i.e. > 5%; *n* = 6). Clustering of mutations using a modified version of PyClone [24] was used to resolve the clonal status of genomic events and to reconstruct the subclonal architecture of tumours. The median number of mutations detected among the cohort of 19 evaluable tumours was 84 (range 26–718). The number of clonal (estimated to be present in every cancer cell) mutations detected (mean 72.2, SD 37.1) was less variable than the number of subclonal mutations (mean 71.4, SD 139.4).

Within the 19 tumours from which at least one sample of adequate tumour purity (> 5% tumour content) was isolated, the karyotype of tumours was characterised using ASCAT [21]. The estimated tumour content of tissue, PDX and cfDNA samples are displayed in Fig. 2. The median weighted genomic instability index of samples, capturing the proportion of the genome that differs from ploidy, was 0.41 (range 0.18–0.91).

Using a list of cancer genes (comprising pan-cancer and pancreatic cancer genes) curated by Bailey et al. [20], we analysed putative driver events within the patient cohort. The majority of patients harboured high-confidence clonal driver mutations within *KRAS* (14/19) and *TP53* (13/19 patients), consistent with previous reports [3, 25]. No subclonal mutations were detected within these genes. SCNAs were also commonly detected at genomic loci containing driver alterations within the 19 tumours with karyotype profiling. There was evidence of clonal loss-of-heterozygosity (LOH) at 9p (encoding *CDKN2A*; 79% of patients, 87% clonal), 17p (*TP53*; 95% of patients, 94% clonal) and 18q (*SMAD4*; 74% of patients, 93% clonal). Gain of the region of chromosome 12p containing the *KRAS* oncogene was seen in 42% of tumours, occurring subclonally in 38% of cases. Other frequently observed SCNAs included loss of *PTEN* (54% of patients, 100% clonal) and *SMARCA4* (58% of patients, 64% clonal).

Next, mutational signatures, reflecting activity of endogenous and exogenous processes during tumour evolution, were characterised across the cohort using deconstructSigs [26]. We observed patterns of common mutational signatures that are consistent with those previously reported, with clock-like mutational processes (e.g. SBS1 and SBS5) underpinning the majority of mutations [27]. Within the tumours of 4 patients harbouring a germline or somatic *BRCA1* or *BRCA2* mutation, a trend towards a higher activity of SBS3 (associated with defects within the homologous recombination DNA repair pathway) was observed (median number of mutations associated with SBS3: 163 vs 24.1, Wilcoxon *p* = 0.096, Additional file 1: Fig. S5). We confirmed the association between a higher number of mutations associated with SBS3 and germline BRCA mutations within the *TCGA* cohort of pancreatic tumours (median number of mutations associated with SBS3: 16.2 vs 3.61, Wilcoxon *p* = 0.029; Additional file 1: Fig. S6). Of note, the same relationship was not observed when comparing tumours with and without loss-of-heterozygosity at these loci. Furthermore, elevated mutation burden and presence of SBS6, a single-base substitution signature associated with mismatch-repair deficiency was observed within patient PANVH4. Further examination of the mutations identified within this tumour revealed a single intergenic mutation, a G > A transition at position 47,739,509 on chromosome 2 (hg19 build; equivalent to position 47,512,370 hg38 build) adjacent to *MSH2*, which has not been reported previously in the literature but might plausibly underpin the SBS6 mutational signature and high mutation burden observed.

### Multiple temporospatial sampling to identify additional genomic variation within ctDNA

To assess the value of ctDNA in monitoring the ongoing evolution of pancreatic cancers, serial blood plasma samples were extracted from each patient. WES was performed on each plasma sample at a median depth of 105 × (range 35–205) and a bespoke analytical pipeline, *ACT-Discover* was developed for the detection of ctDNA and the characterisation of the genomic variation within.

Two-step filtering approaches were applied to detect mutations and SCNAs within ctDNA. Initially, we screened for de novo somatic variants within each sample independently using stringent filters, including a variant allele frequency (VAF) filter of > 5%. Overall, this revealed that a median of 8% (range 0–54%) of mutations detected within ctDNA were not shared with the tissue samples, highlighting that unbiased sampling in the blood can differ substantially from single-sample tissue biopsies that frequently capture a tiny minority of total tumour cells [28].

We subsequently performed an analysis of personalised (i.e. patient-specific) mutations analogous to that carried out in other studies [9, 29–32], in which a lower threshold of evidence was required to detect variants that had also been observed within another tumour sample derived from the same patient (see the “Methods” section). To optimise our approach for the identification of patient-specific mutations within independent samples, we performed a sensitivity analysis for thresholds used in calling mutations (Additional file 1: Figs. S2 & S3, the “Methods” section). This suggested that a mutant read count filter of ≥ 3 reads, combined with a VAF filter of > 1.5% was required to identify true positive mutations while minimising potential false positive calls arising from common sequencing errors.

**Fig. 3:**
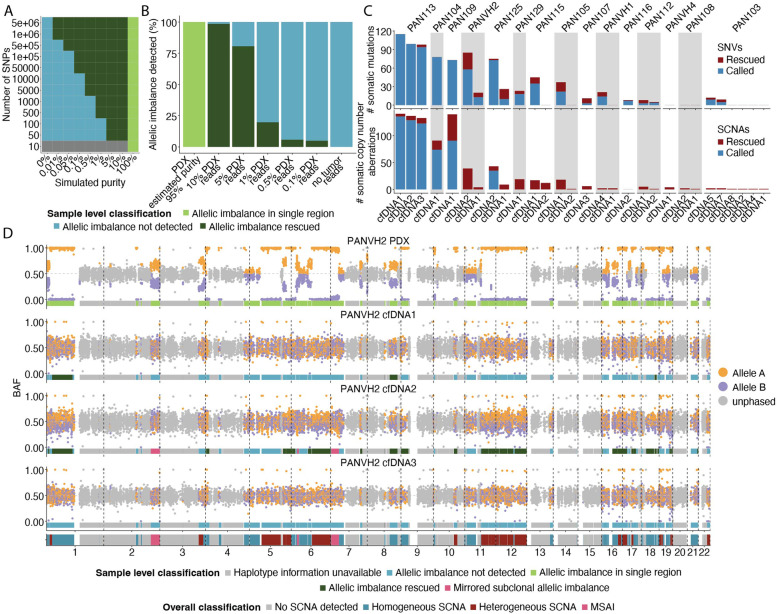
Tumour-informed analysis of somatic mutations and copy number alterations. **A** Simulated data testing the sensitivity of *ACT-Discover* at tumour content between 0 and 10% with differing numbers of simulated SNPs per segment. At a simulated purity of 5–10%, segments containing only 50 SNPs could be rescued using *ACT-Discover*. At a simulated purity of 0.1%, only segments containing 50,000 SNPs or more could be rescued. **B** Downsampling approach testing the sensitivity of *ACT-Discover* in the context of reduced effective tumour content. At simulated purity of ~ 10% (i.e. 10% PDX content), 97% of this allelic imbalance remained detectable. Even at 1% effective purity, 20% of allelic imbalance was detectable, and at 0.1% effective purity, it was still possible to detect allelic imbalance, albeit only 5%. **C** Number of mutations identified within each sample, coloured by whether they were identified using de novo somatic mutation calling or whether they required a tumour-informed approach, and the number of copy number alterations identified within each sample, coloured by whether they were identified using de-novo copy number calling or whether they required a haplotype-informed approach. **D** Copy number profiles of metastasis and three cfDNA samples of patient PANVH2. For each segment, a sample was selected that had the greatest absolute difference of BAF values to 0.5. The SNPs were then coloured based on whether they were greater than 0.5 (orange) or less or equal than 0.5 (purple). This colouring was then used for the SNPs in the segment in all other samples. It was not possible to phase SNPs coloured in grey. Segments where in at least one sample SNPs coloured in purple have BAF values greater than 0.5 can be classified as having mirrored subclonal allelic imbalance. Examples of this can be seen on chromosome 2q and 7p

We then applied the same logic to the detection and analysis of SCNAs. Using ASCAT, genomic segments that were in allelic imbalance within each sample with adequate tumour purity were identified [21]. These segments contained multiple single nucleotide polymorphisms (SNPs), enabling the phasing of parental haplotypes. Using phased SNPs we could then investigate whether allelic imbalance was observed in additional samples, even in those with low tumour purity. Simulated data, whereby segments containing different numbers of SNPs in the context of loss-of-heterozygosity, at different sample tumour fractions (purity), were then queried to establish the sensitivity and specificity of such an approach (Fig. 3A). This indicated that, for example, at purity of 0.1%, a haplotype-aware approach would be able to identify allelic imbalance within a segment containing at least 50,000 SNPs.

Next, we validated these findings using real data from patient tumours. Using a downsampling approach [22], we tested the ability of *ACT-Discover* to identify allelic imbalance with increasingly impure samples (Fig. 3B, Additional file 1: Fig. S7). In data derived from sequencing of the PDX sample (estimated tumour content: 95%) from PAN104, 65% of the genome was identified as being in allelic imbalance. Adding in matched germline reads to simulate an admixed sample of lower purity reduces the fraction of events it is possible for *ACT-Discover* to identify. However, at simulated purity of ~ 10% (i.e. 10% PDX content), 97% of this allelic imbalance remained detectable. Even at 1% simulated purity, 20% of allelic imbalance was detectable, and at 0.1% effective purity, it was still possible to detect allelic imbalance, albeit only 5% of all allelic imbalance. Reassuringly, when we “downsampled” to a simulated purity of 0% (i.e. using 100% germline DNA reads), no allelic imbalance (which would represent a false-positive call) was detected. This suggests that *ACT-Discover* can elucidate substantial information relating to tumour aneuploidy from cfDNA samples with ctDNA fractions of around 1% and can still recover elements of this in samples with even lower tumour content.

We summarise our mutation and SCNA-rescuing approaches in Fig. 3C. We observed a median of 24.5 (range 5–115) mutations within 20 of 47 cfDNA samples, of which a median of 22% (range 0–73%) had been rescued using the patient-informed approach (Fig. 3C). Similarly, we observed a median of 4.5 and mean of 30 (range 1–141) SCNAs within 28 of 47 cfDNA samples. Analysis of the allelic frequencies of phased personalised SNPs within paired samples from within individual patients confirmed this approach enabled the identification of SCNAs at lower thresholds (see the “Methods” section). Strikingly, a median of 100% and mean of 82% (range 3.5–100%) of SCNAs, including every SCNA within 22 cfDNA samples, were only detectable using haplotype phasing, and, as such, would be missed in the absence of *ACT-Discover* (Fig. 3C). Additionally, we also applied *ACT-Discover* to the matched germline samples to ensure we were not overcalling allelic imbalance. Reassuringly, no allelic imbalance was detected.

Figure 3D shows an exemplar case (PANVH2) that illustrates the utility of *ACT-Discover*. Here, the PDX sample enabled inference of parental haplotypes for the 47.4% of the genome that was subject to allelic imbalance. Analysis of groups of SNPs from phased parental alleles within ctDNA uncovered multiple instances of allelic imbalance, at chromosome 1p, 6q, 11q, 12, 17p (containing the *TP53* locus) and 18q within a paired ctDNA sample. The use of an alternative approach, ichorCNA [33], which seeks to identify copy number variation based on observed changes in read depth alone and does not phase SNPs, failed to observe these events, highlighting the added value of *ACT-Discover* (Additional file 1: Fig. S8).

In total, 9 of 16 patients (PAN104, PAN105, PAN109, PAN112, PAN113, PAN115, PAN119, PAN125, PAN129) who had cfDNA samples collected at diagnosis were found to be ctDNA positive based on mutations. Notably, of the 7 who were negative, only 4 patients (PAN110, PAN117, PAN120, PAN121) remained ctDNA-negative during the disease course. Within the 16 patients with evaluable samples at diagnosis, there was a trend that did not meet statistical significance towards a favourable outcome in those without ctDNA detected at that point, consistent with other work in appropriately powered cohorts [34] (*p* = 0.18, Additional file 1: Fig. S9).

### Karyotype intra-tumour heterogeneity throughout the disease course

Using *ACT-Discover*, it was possible within a subset of patients to characterise in detail the evolutionary dynamics of somatic genomic variation, at the level both of point mutations and SCNAs, during the disease course.

**Fig. 4:**
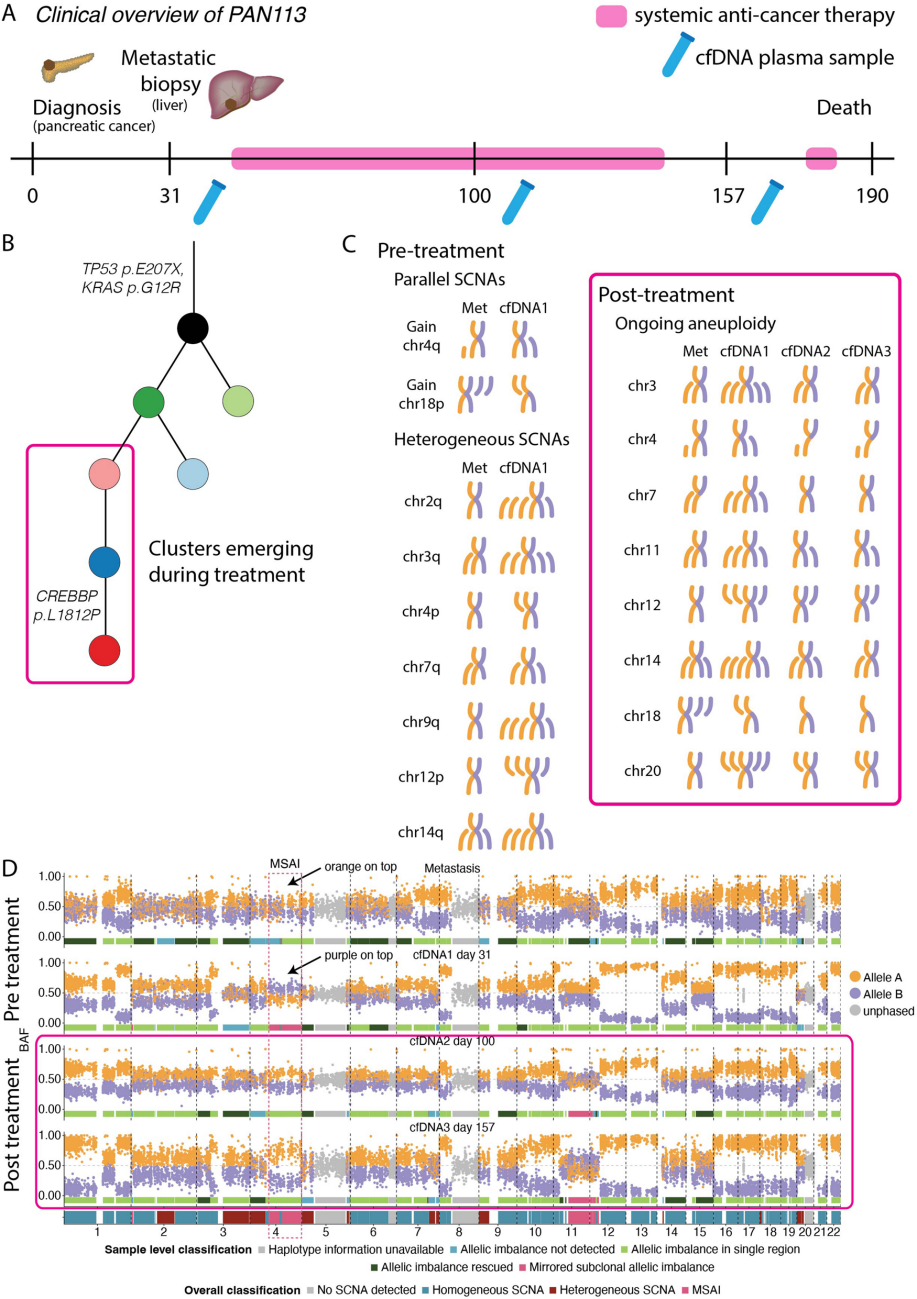
Heterogeneity of somatic copy number alterations in an untreated cancer. **A** Timeline of patient PAN113 showing sample collection and treatment. **B** Phylogenetic tree showing mutation clusters arising prior to, and after, systemic anti-cancer treatment. A putative driver mutation in *CREBBP* arises during treatment. **C** Heterogeneity of somatic copy number alterations (SCNAs). Events occur in parallel, potentially indicative of convergent evolution, or are heterogeneous between a metastatic sample and cfDNA extracted at the same time point. Ongoing heterogeneity is detected over time in subsequent cfDNA samples. **D** Copy number profiles of metastasis and three cfDNA samples of patient PAN113

One such patient, PAN113, presented with metastatic pancreatic cancer. Thirty-one days following diagnosis, and prior to the patient receiving systemic anti-cancer therapy, a biopsy of the metastatic lesion (estimated tumour content of 44%) was obtained alongside a concomitant cfDNA sample with estimated ctDNA content of 70%, according to ASCAT (Fig. 4A). It was therefore possible to reconstruct a detailed estimate of tumour karyotype from both samples. Each sample revealed substantial aneuploidy, with 51% and 82% of the genome in allelic imbalance within the metastatic sample and ctDNA, respectively. However, substantial copy number heterogeneity, affecting 22% of the genome, was observed between the karyotypes derived from the metastatic biopsy and the ctDNA sample. Notably, mirrored subclonal allelic imbalance (MSAI; where the maternal allele is gained or lost in one subclone of a tumour, but the paternal allele is gained or lost in another independently) was identified at two genomic loci: part of chromosome 4q (containing the tumour suppressor *FAT1*) and a focal segment on the p arm of chromosome 2 (Fig. 4C, D). These events are suggestive of parallel evolution, converging upon disruption to specific genomic loci, in this case, events at 4q and 2p. Furthermore, this highlights the presence of at least two distinct subclones at the point of sampling and prior to systemic treatment, one of which was not biopsied but was capable of shedding an amount of ctDNA that exceeded that of the subclone that was sampled at diagnosis.

Furthermore, in PAN113, serial ctDNA biopsies were extracted on days 50 and 137 following diagnosis (Fig. 4A). The ctDNA content of these samples was similarly high, at 37% and 76%, respectively. These samples were taken during systemic anti-cancer treatment with the agents gemcitabine and paclitaxel, and further evolution of mutations and SCNAs was observed. For example, a tumour subclone was detected at day 137 containing a de novo mutation within the cancer gene *CREBBP* that was not detected in either the metastatic biopsy or within the previous two cfDNA samples (Fig. 4B). Within this subclone that may have emerged in response to treatment, mutations were also detected within the genes *AKAP9* and *ACSL6*, both of which are listed in the COSMIC cancer gene census [35]. Importantly, there was evidence of further karyotype remodelling during treatment (Fig. 4C, D). MSAI was observed on chromosome arms 11q (containing a number of cancer genes including *ATM* and *KMT2A*) and 12p, suggestive of ongoing clonal evolution and potential selection of SCNAs during treatment in this patient (Fig. 4C, D). Other SCNA heterogeneity was observed at sites of chromosomes 3, 4, 7, 14, 18 and 20.

It was also possible to compute phylogenetic trees for a total of 6 patients (Additional file 1: Fig. S10) and to detect copy number information for a total of 15 cases using liquid biopsy samples (Additional file 1: Figs. S11-S25).

For case PANVH2, in whom one cfDNA sample containing sufficient ctDNA to study SCNAs was extracted during follow-up, differences in copy number profile were observed across multiple chromosomes, including MSAI at chromosome 2q, 6p and 7p. Similarly, in PAN108 focal segments of MSAI were observed in the cfDNA samples on chromosomes 17 and 19. Further heterogeneity was observed in SCNAs between tissue biopsies and ctDNA samples within PAN104 and PAN109; of note, the ctDNA from PAN109 was extracted on the same day as the metastasis was sampled.

In total, using an approach that classified genomic segments according to whether allelic imbalance was homogenous (i.e. consistent across samples with sufficient tumour content to detect it at high confidence) or heterogeneous (i.e. there were instances of karyotype heterogeneity across samples), 13 of 15 patients demonstrated evidence of karyotype heterogeneity with a median of 26.6% (range 0–91.2%) of the genome affected by allelic imbalance found to be heterogeneous across samples in a patient. Importantly, this feature was strongly correlated to the estimated tumour content of cfDNA samples based on the mean clonal VAF (Additional file 1: Figs. S4, S26, the “Methods” section). This highlights that karyotype heterogeneity is relatively common in pancreatic cancer, and that our power to determine its ubiquity is intimately linked to the extent of sampling (in this case, it is possible that lack of tumour content masks the discovery of spatio-temporal inter-sample heterogeneity).

## Discussion

Liquid biopsy and the analysis of cell-free DNA has emerged as a promising biomarker for patient stratification and the detection of minimal residual disease [9, 36, 37]. It has also shown convincing utility in studies of tumour evolution, such as tracking of phylogenetic clones using point mutations over time [9]. However, it is clear that substantial functionally impactful variation occurs at the level of somatic copy number alterations. The ability to detect such events through liquid biopsy could represent an important adjunct to studies of tumour evolution.

To that end we have developed a novel approach, *ACT-Discover*, which leverages allele-specific haplotype phasing from a tumour, PDX or high-purity cfDNA sample to identify heterogeneity of allelic imbalance between samples taken across time and space, thereby enabling exploration of ongoing tumour evolution.

Insights into pancreatic cancer evolution prior to and in response to treatment are urgently required given that 70% of patients die within one year of diagnosis [2]. In a small cohort of patients with advanced pancreatic cancer, we have used *ACT-Discover* to reveal karyotype heterogeneity both before and during treatment across multiple patients with late-stage disease, suggestive of ongoing genetic remodelling that would not be otherwise detected.

In our small cohort, we observe homogeneity among driver mutations such as within *KRAS* and *TP53*, which in isolation are supportive of the model of pancreatic cancer evolution that describes a punctuated course with early, clonal events. However, within one case, we find significant pre and post-treatment karyotype heterogeneity, and evidence within ctDNA of a putative driver mutation within the tumour suppressor gene *CREBBP* arising through treatment over time. Analysis of allele-specific haplotype profiles reveals MSAI affecting disparate genomic loci, with 12 instances of this phenomenon occurring across four cases. This might indicate parallel evolution and selection whereby there is ongoing optimisation of clonal fitness.

Within our study, which was limited by the extent of ctDNA shedding from each tumour, and the resultant tumour fraction detected within cfDNA, it was only possible to investigate allelic imbalance using *ACT-Discover* within 16 patients. Within this subset of patients, we identified an element of karyotypic heterogeneity within each case, highlighting the potential additional information that might be conferred by *ACT-Discover*.

*ACT-Discover* is not the first approach to search for SNCAs within cfDNA. Indeed, other approaches leverage the subtle differences in read depth or B-allele frequencies suggestive of underlying SCNAs in order to increase assay sensitivity [38–40]. *ACT-Discover* leverages tumour-informed allele-specific SCNA detection to explore karyotype heterogeneity during tumour evolution. Future studies should consider this dimension where possible, and need only be limited by the extent of aneuploidy within tumours, the fraction of the genome sampled (whole-genome sequencing might augment this approach), the ctDNA fraction within the blood (which can be substantial, particularly in late-stage disease), as well as multiple sampling.

To leverage the haplotype-phasing approach of *ACT-Discover*, at least one sample of sufficient tumour purity to identify parental haplotypes within areas of allelic imbalance, particularly loss-of-heterozygosity, is required. Alternatively, orthogonal methods for phasing, for example, through long-read sequencing, could be applied. Of note, in this study, 8 PDXs had been curated. PDXs provide extremely pure tumours, and in our study enabled haplotyping of substantially greater fractions of the genome than would otherwise have been available. This highlights an important auxiliary function of PDXs [41].

Notwithstanding the limitations of the cohort size and ctDNA detection methodology presented here, through the use of whole-exome sequencing of liquid biopsy specimens, we detected ctDNA within the blood of 9 out of 16 patients at the time of metastatic biopsy. A trend towards worse outcomes was observed within these patients. Importantly, the approach used in this study was not optimised for ctDNA detection and sensitivity in comparison with other approaches [9, 36, 37] (such as multiplex droplet digital PCR), and so it is likely that the 7 patients in whom ctDNA was not detected might include “false-negative” cases, where ctDNA was present but below the limit of detection. Taken with other studies, which have identified ctDNA within a plurality of patients with pancreatic adenocarcinoma, this suggests ctDNA might be a biomarker in pancreatic cancer patients [42].

One limitation of this work is that through using conventional whole-exome sequencing of ctDNA, we are restricted to the analysis of samples with very high tumour content in the blood. Therefore, it is likely we have underestimated the overall degree of karyotype heterogeneity among the cohort. Furthermore, future work should seek to understand the extent of SCNA heterogeneity throughout the disease course, from pre-cancer lesions to primary and metastatic disease. This would likely only be possible with approaches that consider the whole genome, or individual sites at much greater depth.

## Conclusions

Our findings underscore the importance of sampling approaches across time and space in cancer, as well as the importance of ctDNA-based approaches in documenting the course of tumour evolution, and highlight intra-tumour heterogeneity within late-stage pancreatic cancer.

## Supplementary Information

**Additional file 1.** All supplementary figures.

**Additional file 2.** Supplementary table with purity information for all samples.

## Data Availability

Raw sequencing reads are available on EGA under EGAS00001007077 (https://ega-archive.org/studies/EGAS00001007077) for all tissue, cfDNA and matched blood normal samples [43]. All processed data and code to reproduce figures can be viewed here: https://github.com/McGranahanLab/ACTdiscover-figure-code-2023 [44]. *ACT-Discover* can be found here: https://github.com/McGranahanLab/ACTdiscover [45].
