# Supplementary material for: ACT-Discover: identifying karyotype heterogeneity in pancreatic cancer evolution using ctDNA
Source: Genome Med. 2023 Apr 20;15:27. doi: 10.1186/s13073-023-01171-w (PMC10120117; doi:10.1186/s13073-023-01171-w)
Supplement: Supplementary file 1 — Additional file 1. All supplementary figures. [file 13073_2023_1171_MOESM1_ESM.pdf]

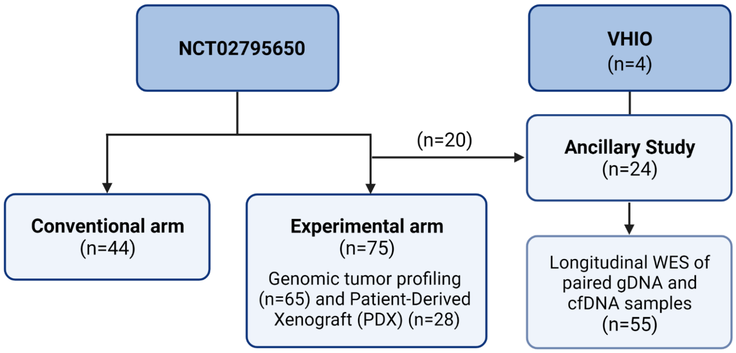

Fig. S1. Consort diagram. The overview of the study arms is shown with a subset of the patients within the experimental arm (ancillary study) subjected to WES and analysed.

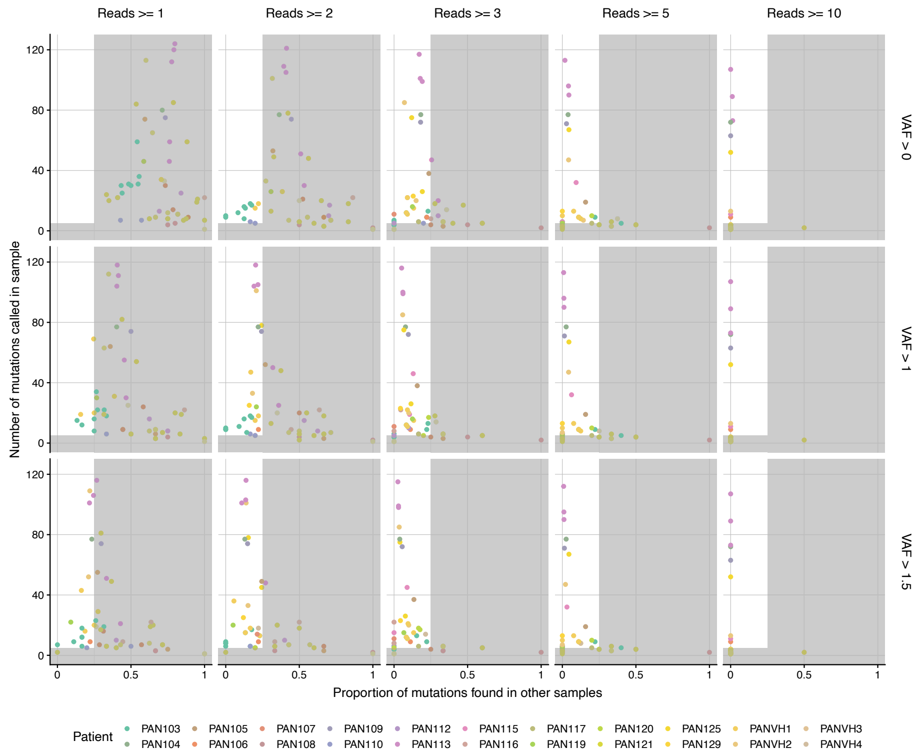

Fig. S2. Overview of effect of different filters on mutation load and false positive rate. The figure shows the number of mutations called in each cfDNA sample on the y-axis and highlights the proportion of such mutations found in cfDNA samples of other patients on the x-axis. The columns highlight the read count filter used while the rows show the VAF filter used to define a mutation as present. The gray area in each plot highlights samples that have less than 5 mutations called or where more than 25% of mutations are found in other samples.

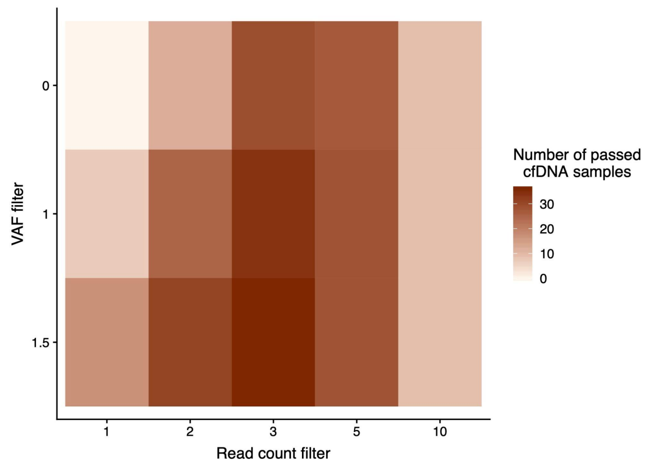

Fig. S3. Number of cfDNA samples retained for each set of filters.

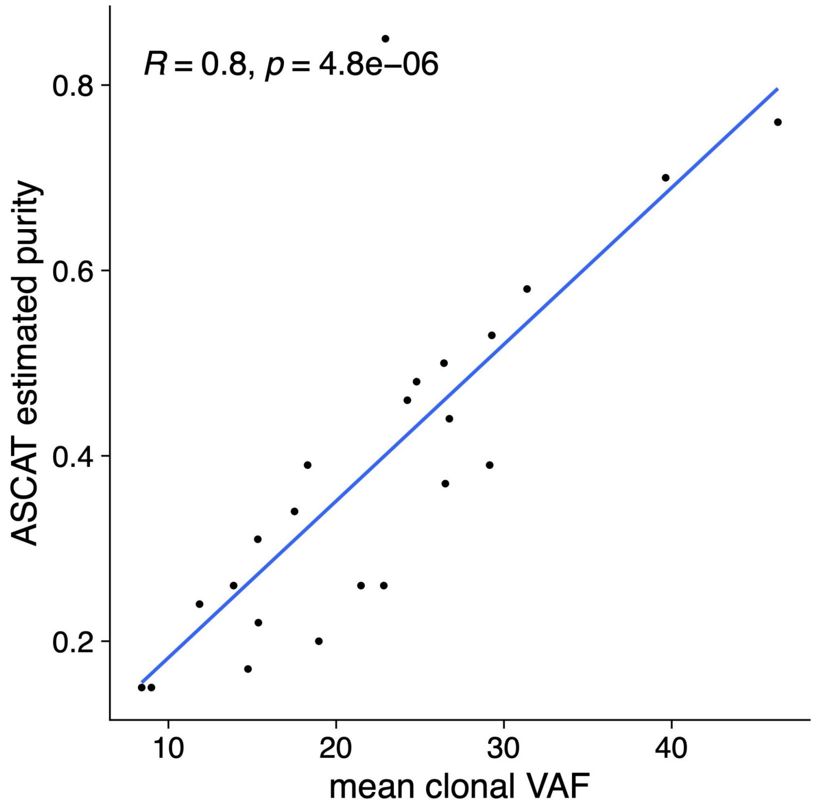

Fig. S4. The purity estimated from ASCAT for tissue samples as well as 6 cfDNA samples is highly correlated with the mean VAF of clonal mutations within these samples.

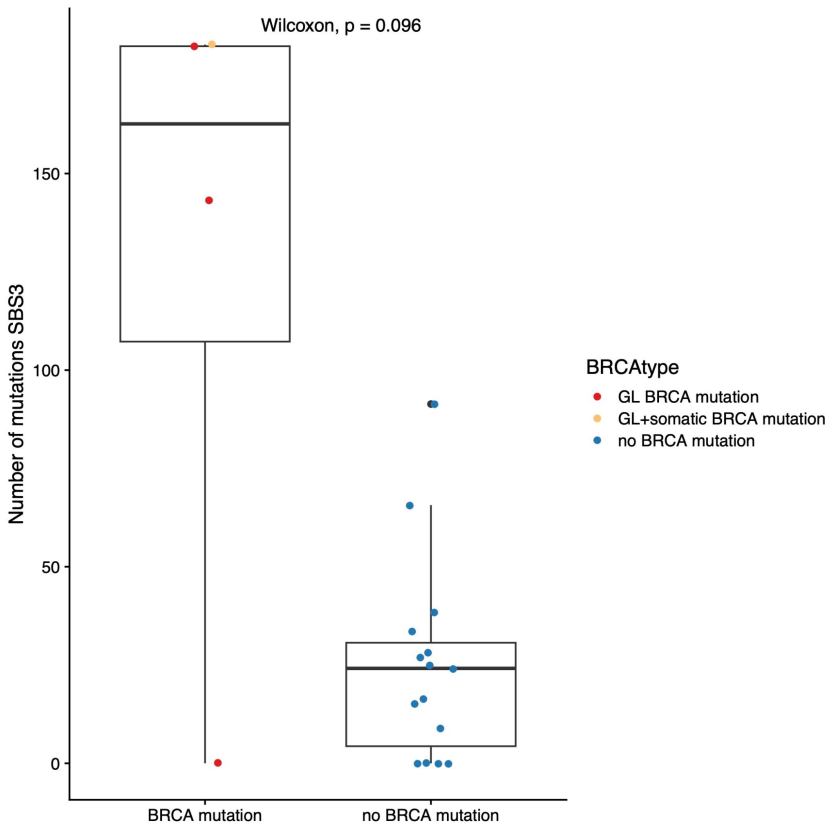

Fig. S5. Comparison of number of mutations associated with SBS3 in tumors with mutations in BRCA1/2 and those without any BRCA mutations within the cohort.

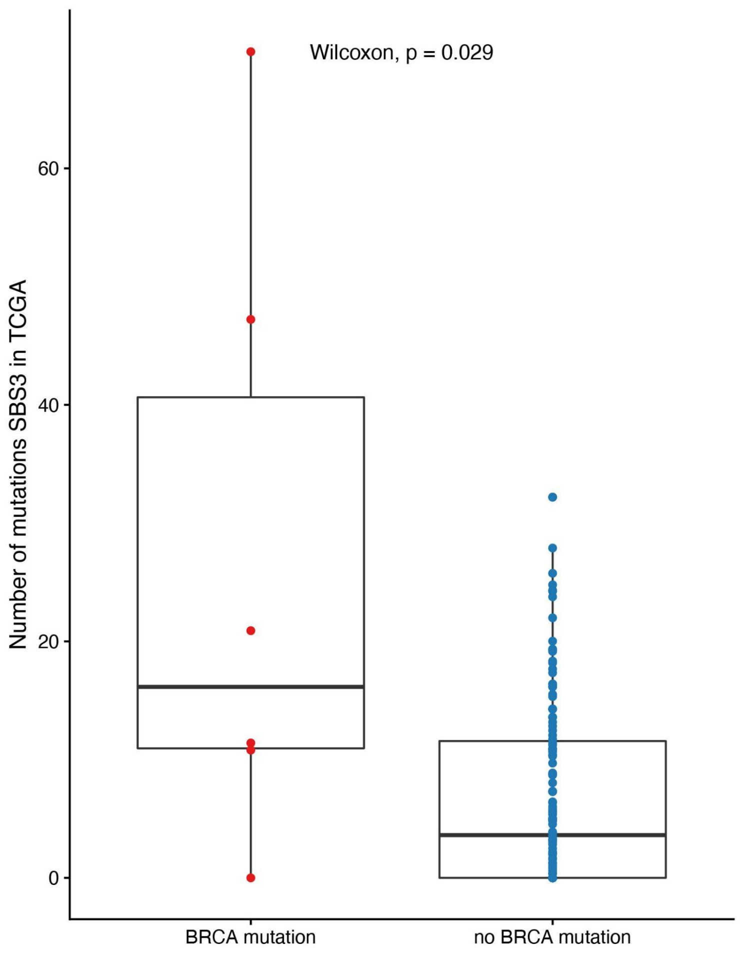

Fig. S6. Comparison of number of mutations associated with SBS3 in tumors with mutations in BRCA1/2 and those without any BRCA mutations within TCGA.

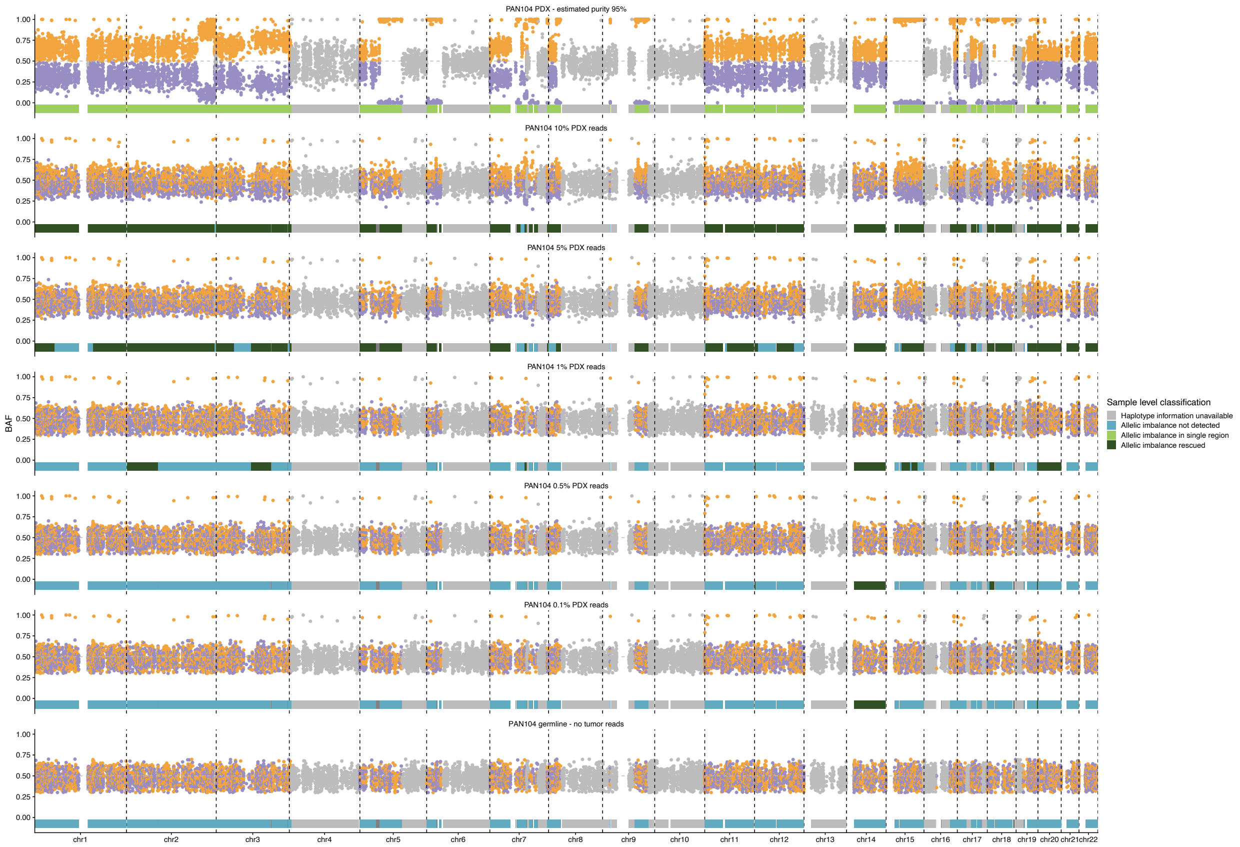

Fig. S7. Downsampling approach testing the sensitivity of *ACT-Discover* in the context of reduced effective tumour content. All SNPs in segments across the genome are shown at different effective purities. The summary tracks for each sample highlight for which segments allelic imbalance could be rescued.

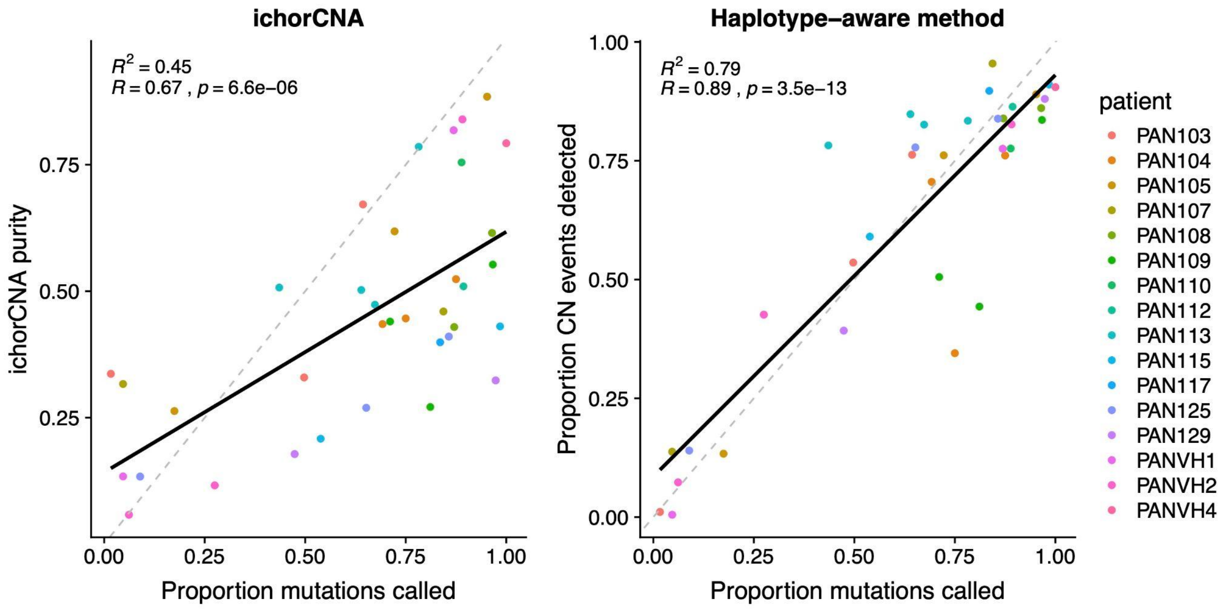

Fig. S8. Comparison of ichorCNA and the haplotype-aware method.

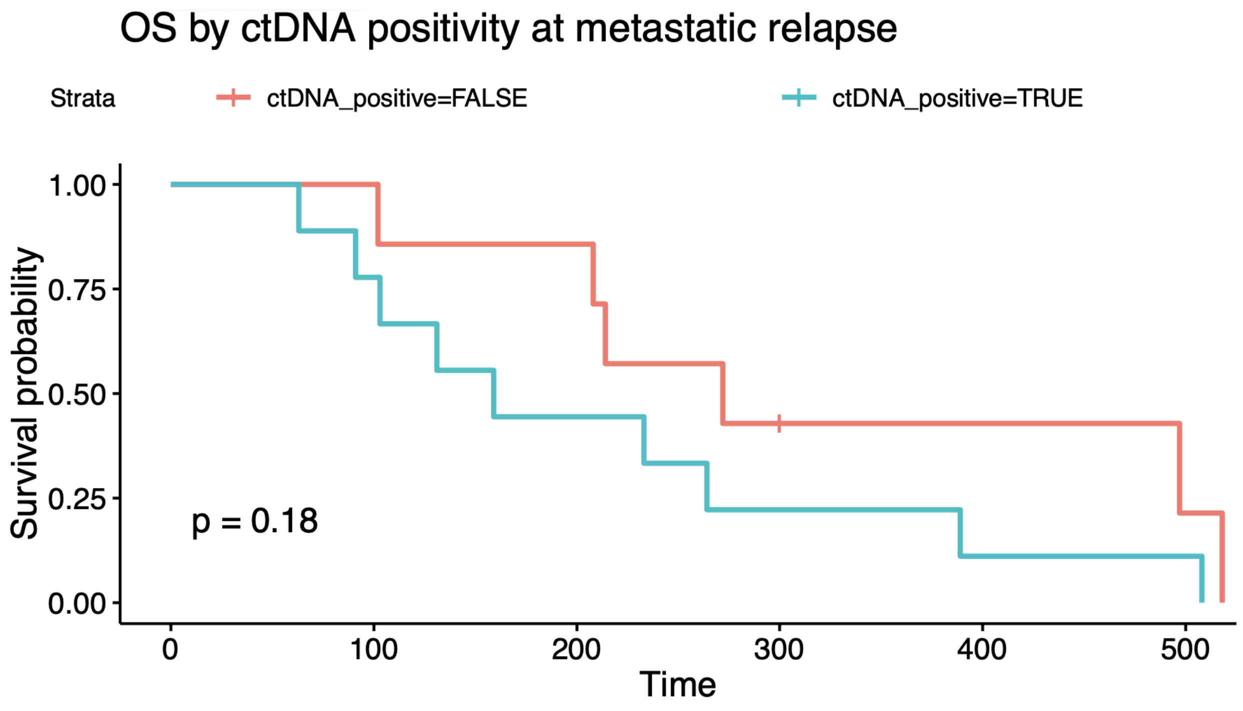

Fig. S9. Survival of patients split by ctDNA presence at diagnosis.

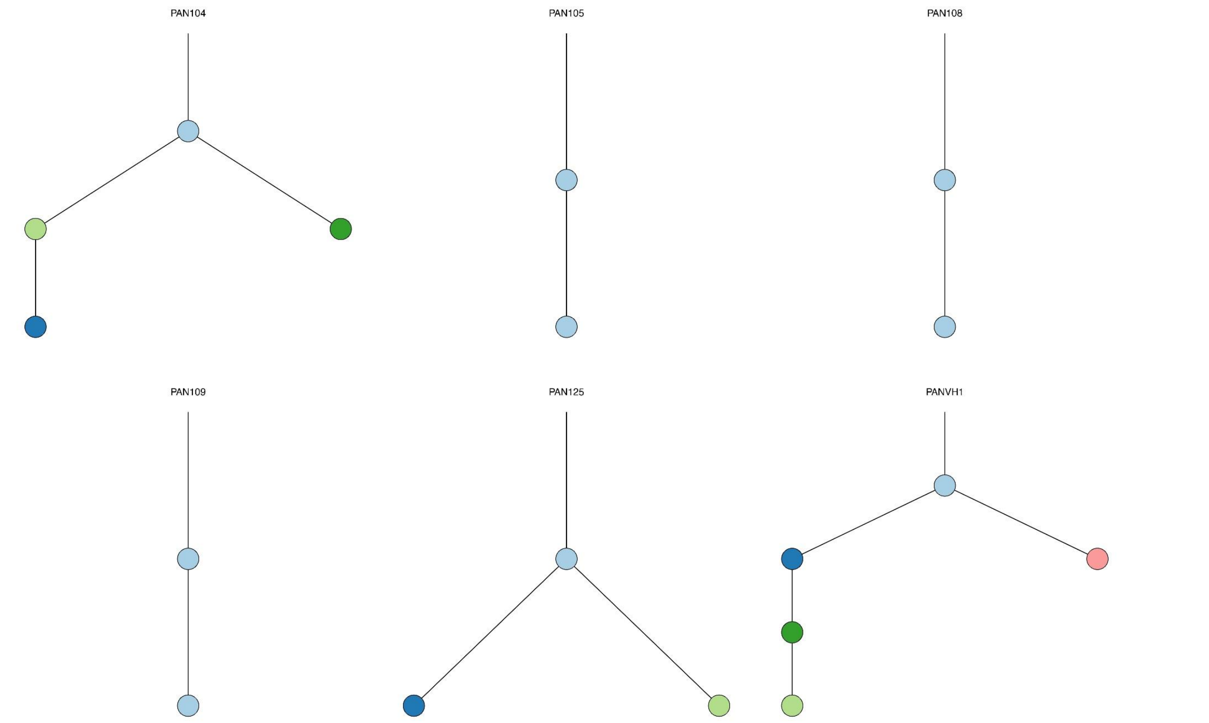

Fig. S10. Phylogenetic trees for 6 patients with multiple samples with sufficient tumour purity.

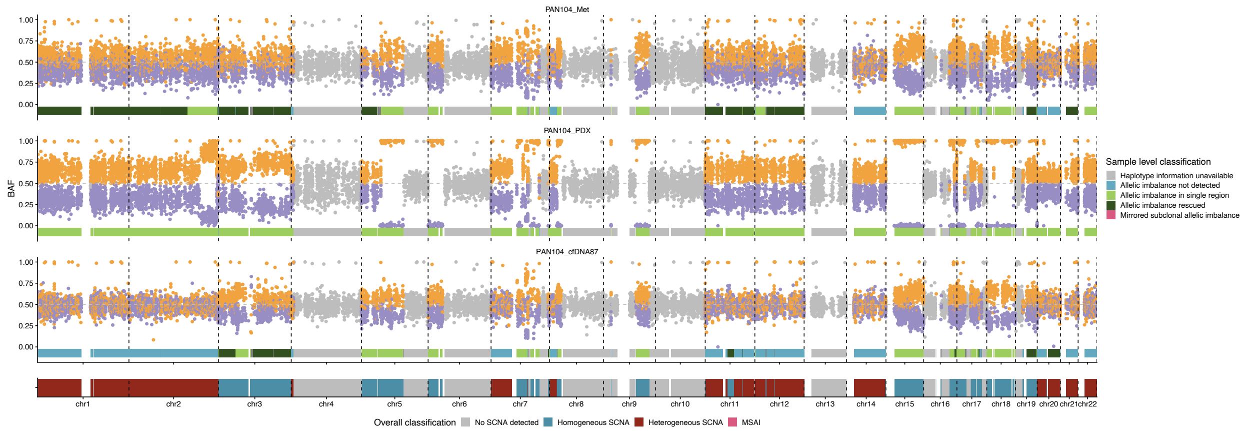

Fig. S11. Copy number profiles of metastasis, PDX and a cfDNA sample of patient PAN104. For each sample, a track at the bottom shows which segments were uniquely called, rescued, or not detected. The summary track at the bottom shows which segments were homogeneous or heterogeneous across all samples of the patient.

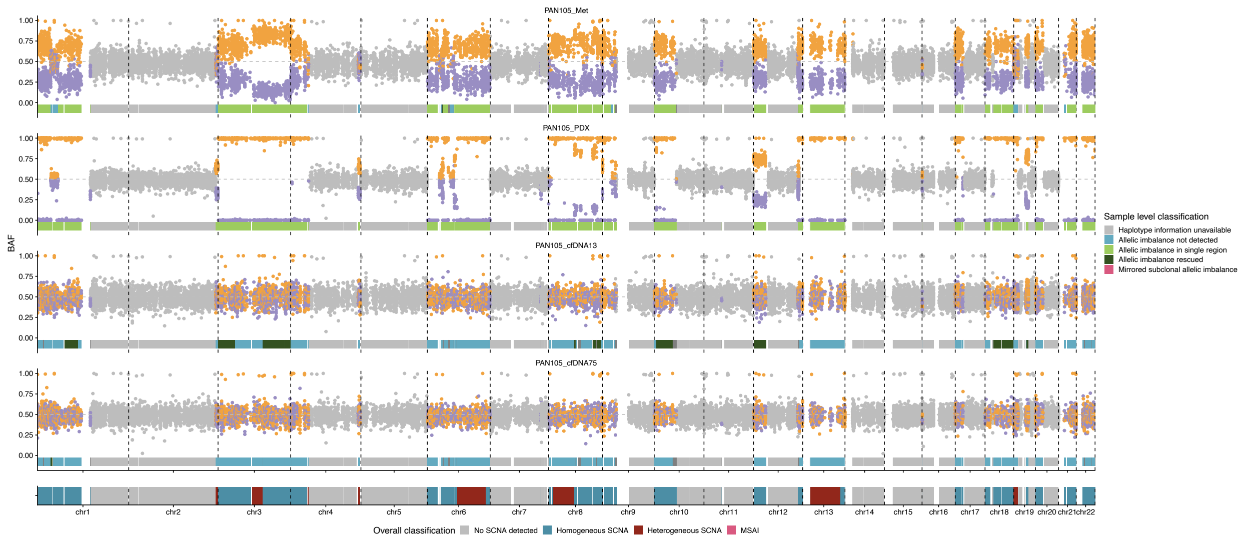

Fig. S12. Copy number profiles of metastasis, PDX and two cfDNA samples of patient PAN105. For each sample, a track at the bottom shows which segments were uniquely called, rescued, or not detected. The summary track at the bottom shows which segments were homogeneous or heterogeneous across all samples of the patient.

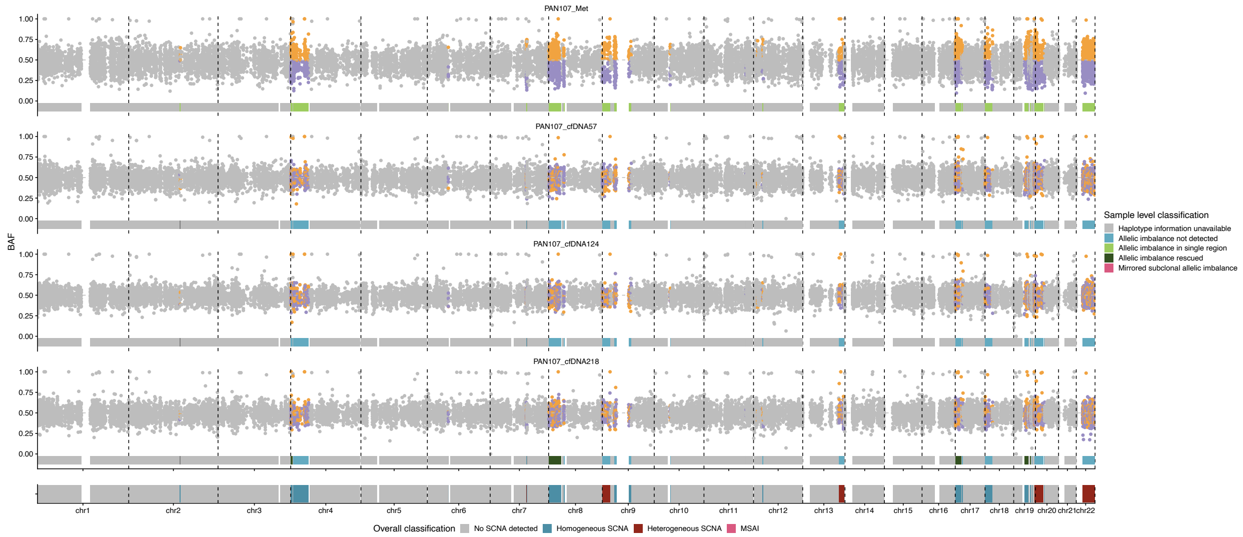

Fig. S13. Copy number profiles of metastasis and three cfDNA samples of patient PAN107. For each sample, a track at the bottom shows which segments were uniquely called, rescued, or not detected. The summary track at the bottom shows which segments were homogeneous or heterogeneous across all samples of the patient.

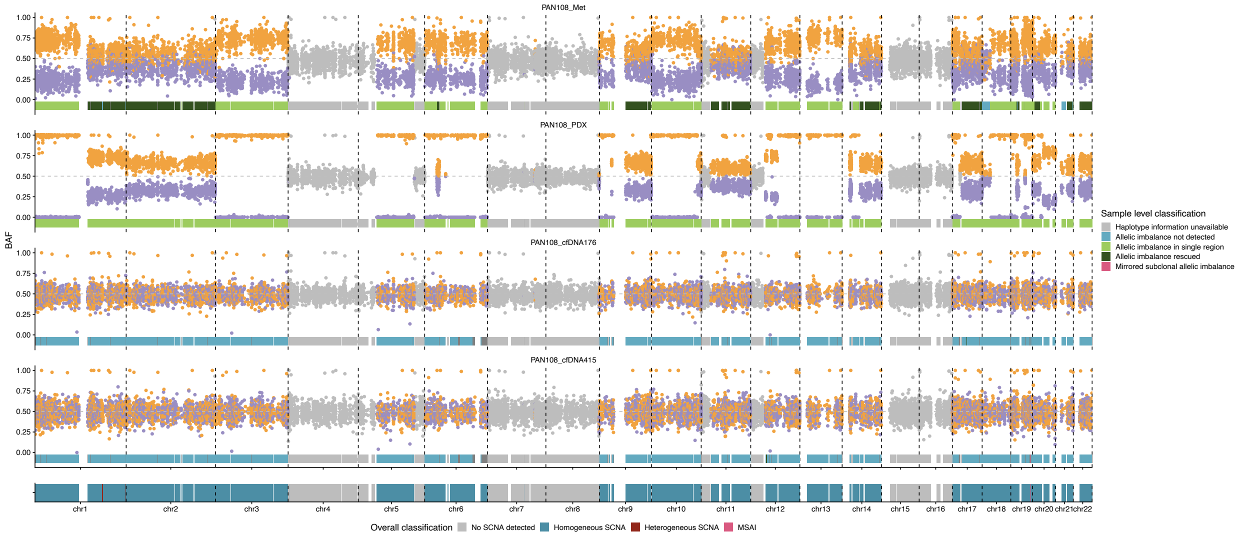

Fig. S14. Copy number profiles of metastasis, PDX and two cfDNA samples of patient PAN108. For each sample, a track at the bottom shows which segments were uniquely called, rescued, or not detected. The summary track at the bottom shows which segments were homogeneous or heterogeneous across all samples of the patient.

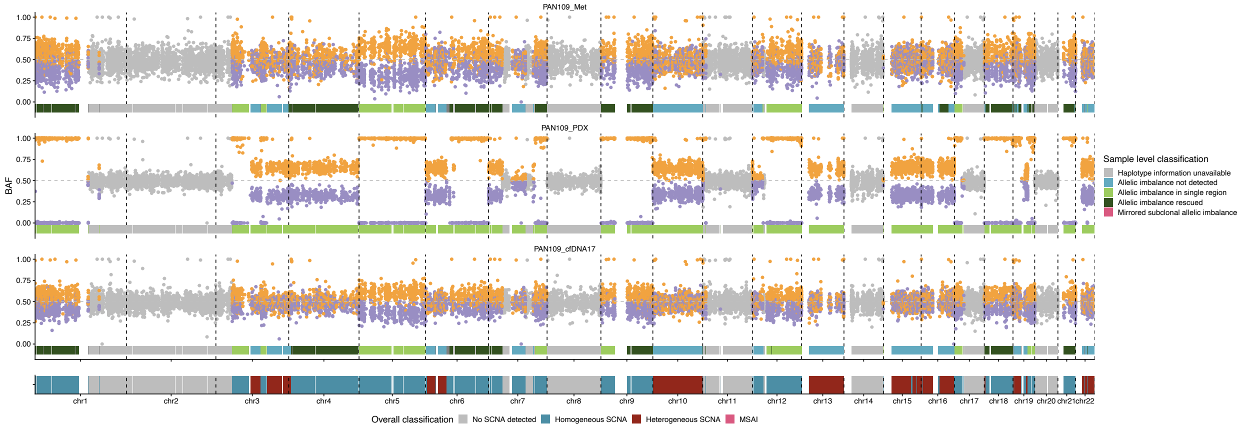

Fig. S15. Copy number profiles of metastasis, PDX and a cfDNA sample of patient PAN109. For each sample, a track at the bottom shows which segments were uniquely called, rescued, or not detected. The summary track at the bottom shows which segments were homogeneous or heterogeneous across all samples of the patient.

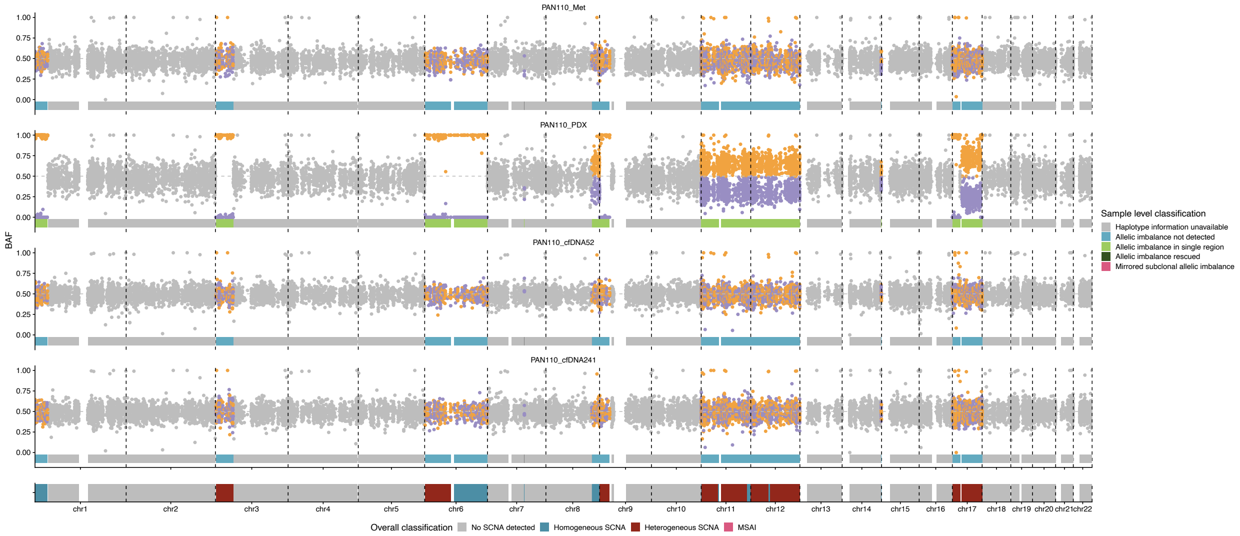

Fig. S16. Copy number profiles of metastasis, PDX and two cfDNA samples of patient PAN110. For each sample, a track at the bottom shows which segments were uniquely called, rescued, or not detected. The summary track at the bottom shows which segments were homogeneous or heterogeneous across all samples of the patient.

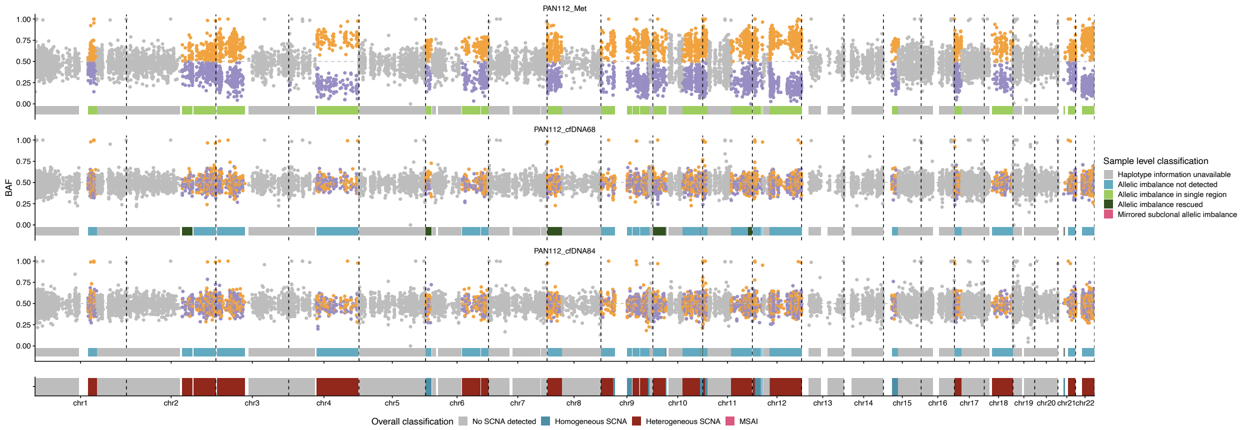

Fig. S17. Copy number profiles of metastasis and two cfDNA samples of patient PAN112. For each sample, a track at the bottom shows which segments were uniquely called, rescued, or not detected. The summary track at the bottom shows which segments were homogeneous or heterogeneous across all samples of the patient.

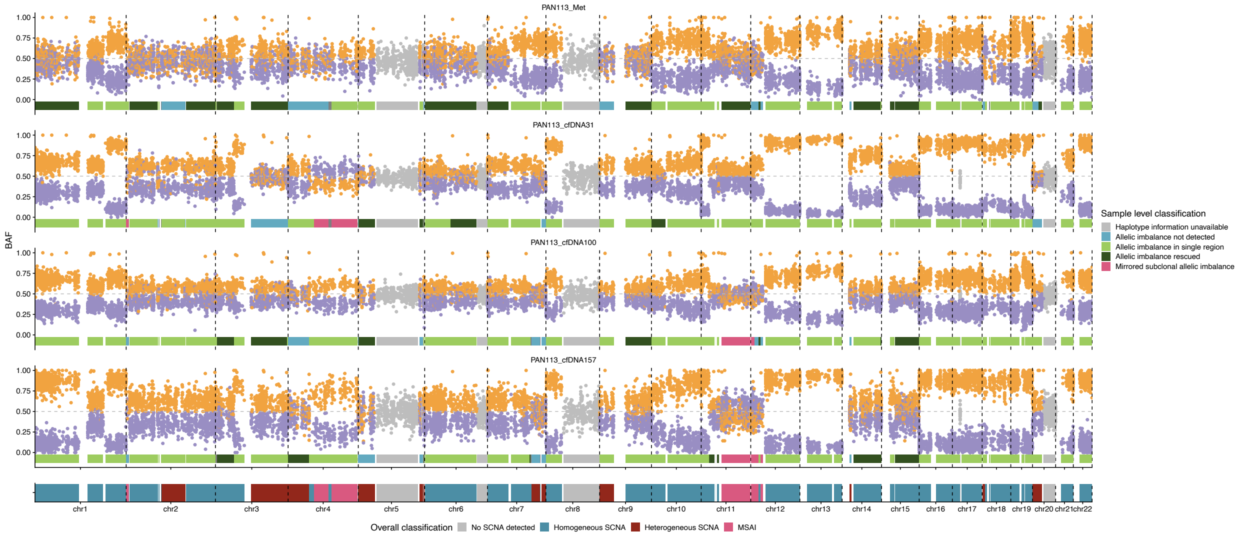

Fig. S18. Copy number profiles of metastasis and three cfDNA samples of patient PAN113. For each sample, a track at the bottom shows which segments were uniquely called, rescued, or not detected. The summary track at the bottom shows which segments were homogeneous or heterogeneous across all samples of the patient.

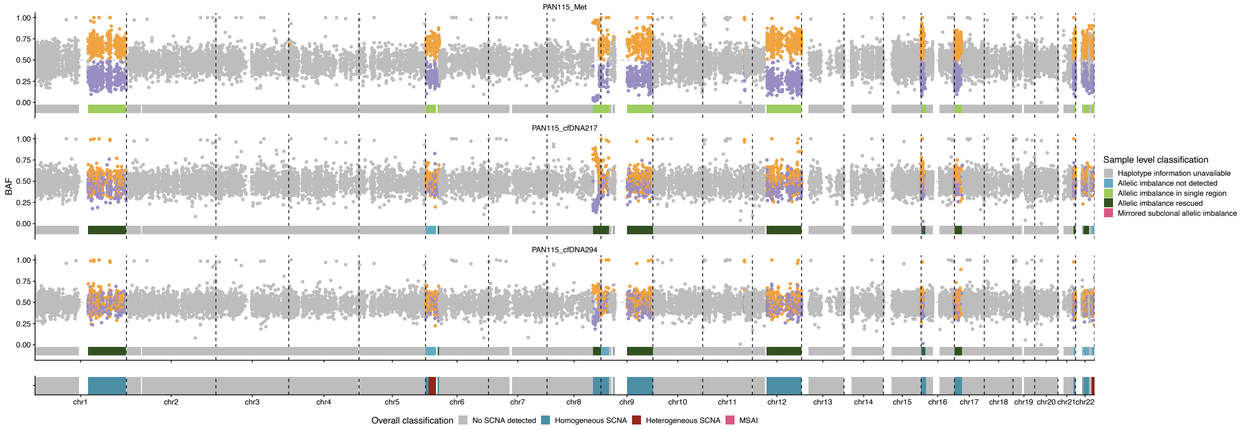

Fig. S19. Copy number profiles of metastasis and two cfDNA samples of patient PAN115. For each sample, a track at the bottom shows which segments were uniquely called, rescued, or not detected. The summary track at the bottom shows which segments were homogeneous or heterogeneous across all samples of the patient.

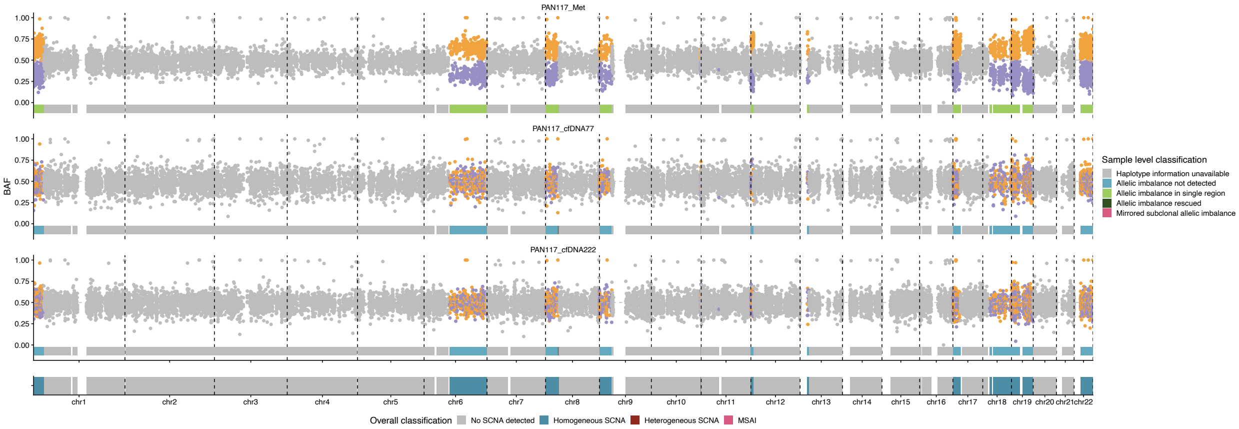

Fig. S20. Copy number profiles of metastasis and two cfDNA samples of patient PAN117. For each sample, a track at the bottom shows which segments were uniquely called, rescued, or not detected. The summary track at the bottom shows which segments were homogeneous or heterogeneous across all samples of the patient.

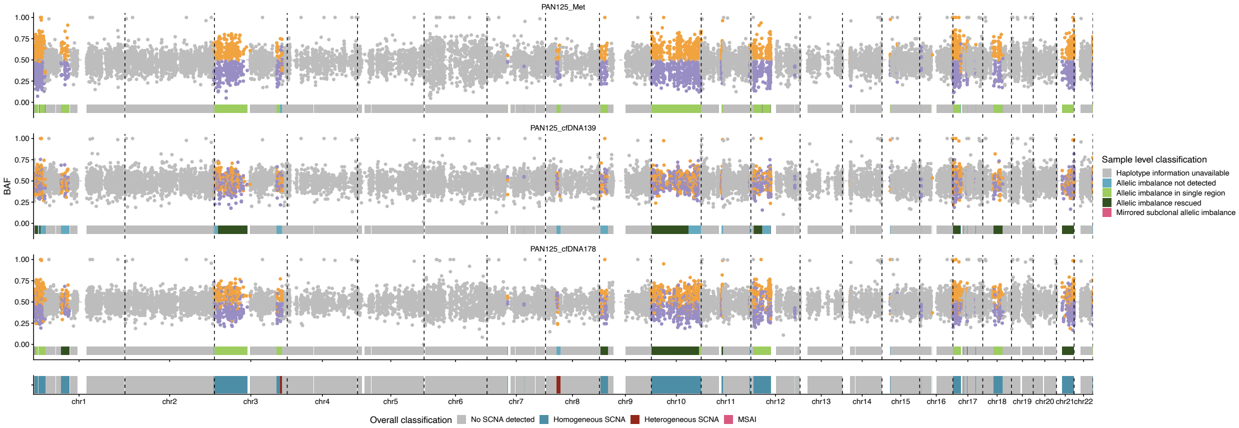

Fig. S21. Copy number profiles of metastasis and two cfDNA samples of patient PAN125. For each sample, a track at the bottom shows which segments were uniquely called, rescued, or not detected. The summary track at the bottom shows which segments were homogeneous or heterogeneous across all samples of the patient.

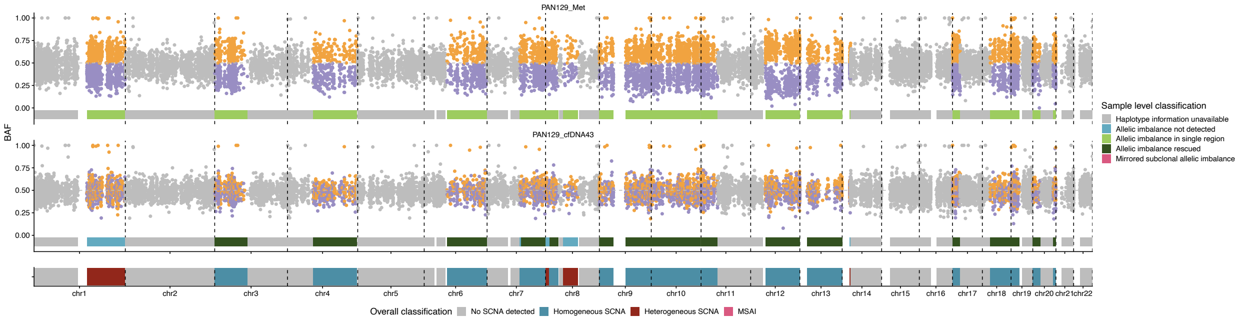

Fig. S22. Copy number profiles of metastasis and a cfDNA sample of patient PAN129. For each sample, a track at the bottom shows which segments were uniquely called, rescued, or not detected. The summary track at the bottom shows which segments were homogeneous or heterogeneous across all samples of the patient.

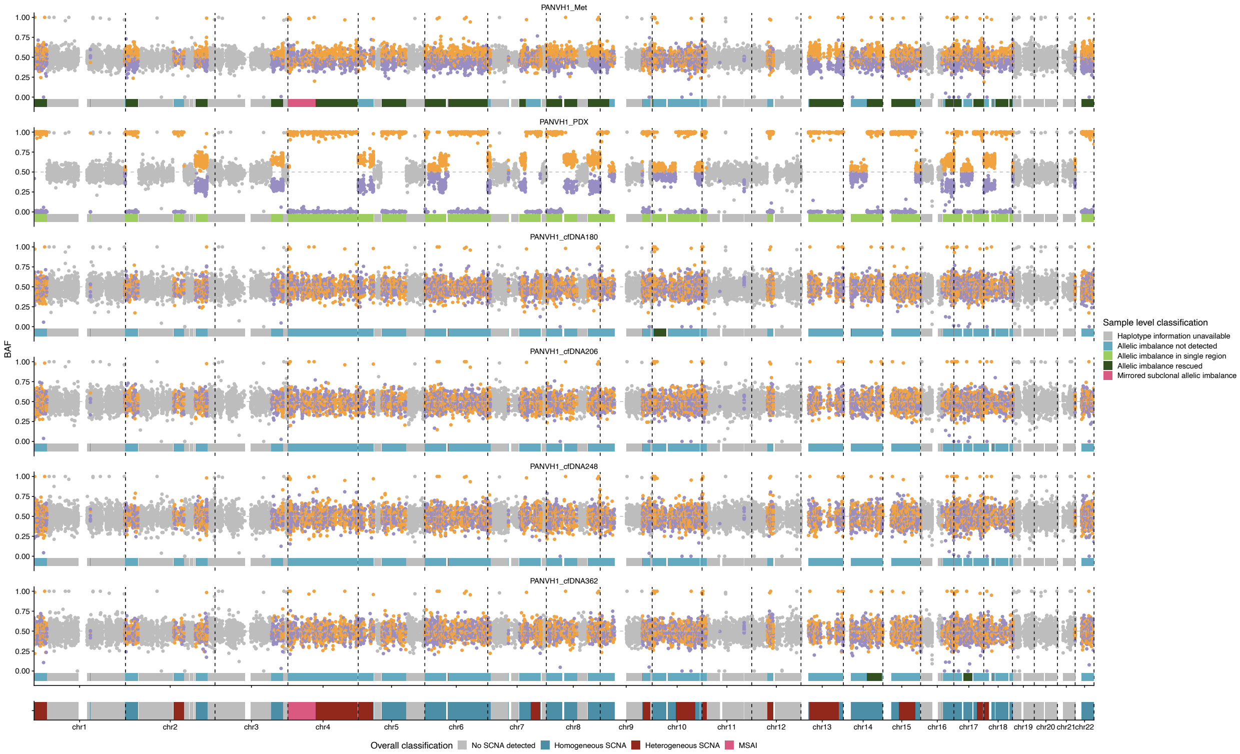

Fig. S23. Copy number profiles of metastasis, PDX and four cfDNA samples of patient PANVH1. For each sample, a track at the bottom shows which segments were uniquely called, rescued, or not detected. The summary track at the bottom shows which segments were homogeneous or heterogeneous across all samples of the patient.

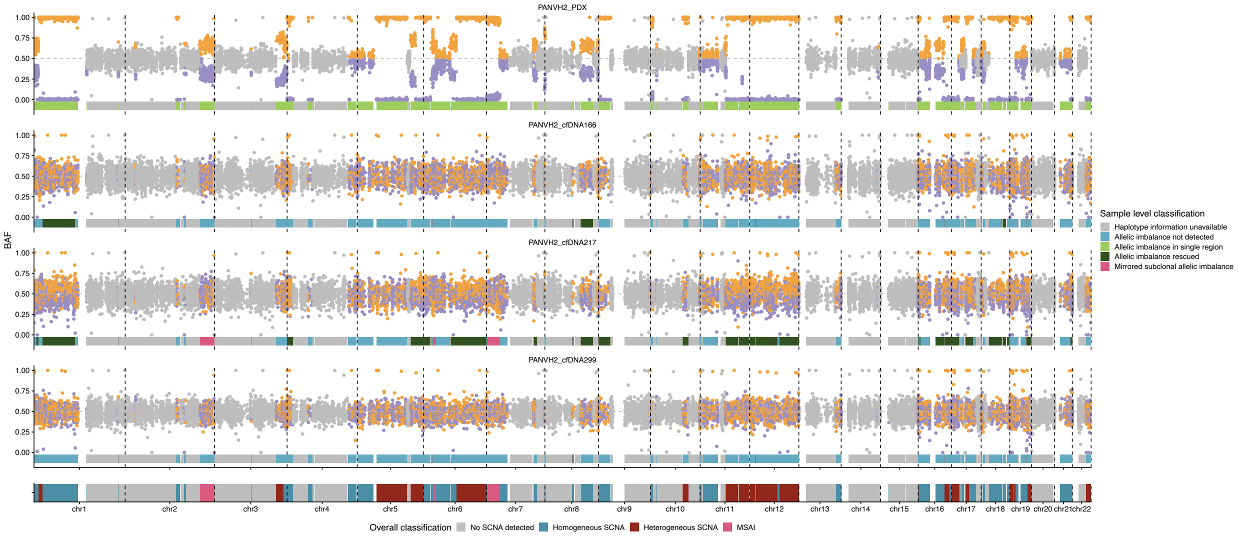

Fig. S24. Copy number profiles of metastasis and three cfDNA samples of patient PANVH2. For each sample, a track at the bottom shows which segments were uniquely called, rescued, or not detected. The summary track at the bottom shows which segments were homogeneous or heterogeneous across all samples of the patient.

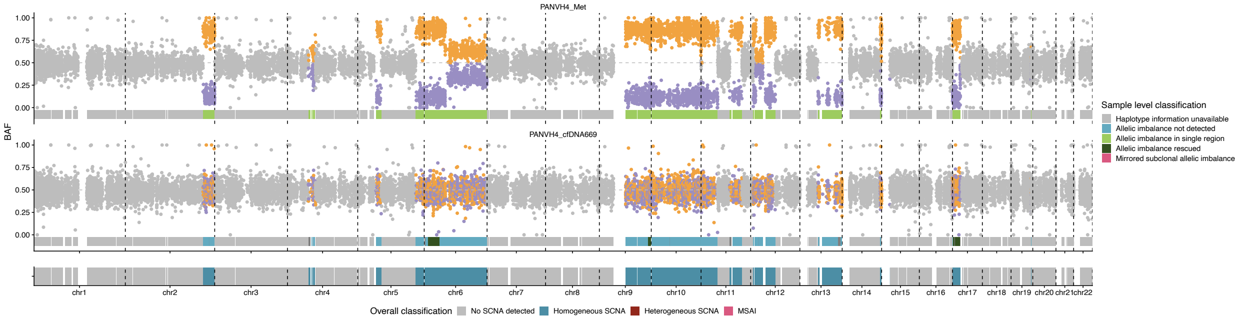

Fig. S25. Copy number profiles of metastasis and a cfDNA sample of patient PANVH4. For each sample, a track at the bottom shows which segments were uniquely called, rescued, or not detected. The summary track at the bottom shows which segments were homogeneous or heterogeneous across all samples of the patient.

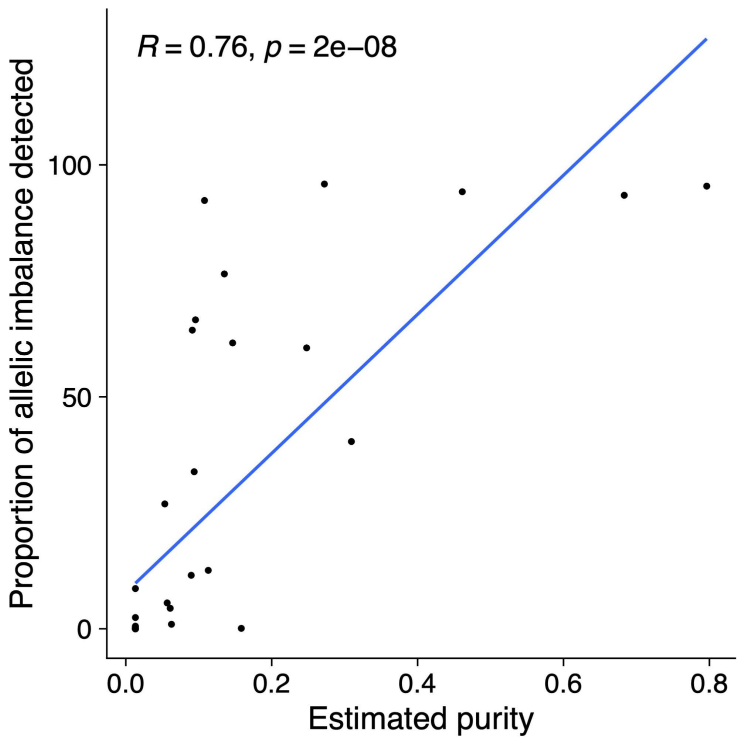

Figure S26. The proportion of allelic imbalance detected in each cfDNA sample is correlated with the estimated purity calculated for each cfDNA sample from the mean clonal VAF.
